# Carbon nanotubes: a powerful bridge for conductivity and flexibility in electrochemical glucose sensors

**DOI:** 10.1186/s12951-023-02088-7

**Published:** 2023-09-07

**Authors:** Tianyi Yuwen, Danting Shu, Hanyan Zou, Xinrui Yang, Shijun Wang, Shuheng Zhang, Qichen Liu, Xiangxiu Wang, Guixue Wang, Yuchan Zhang, Guangchao Zang

**Affiliations:** 1 Institute of Life Science, and Laboratory of Tissue and Cell Biology, Lab Teaching & Management Center, Chongqing Medical University, Chongqing, 400016 China; 2 Key Laboratory of Biorheological and Technology of Ministry of Education, State and Local Joint Engineering Laboratory for Vascular Implants, Bioengineering College of Chongqing University, Chongqing, 400030 China; 3 JinFeng Laboratory, Chongqing, 401329 China; 4 Chongqing Institute for Food and Drug Control, Chongqing, 401121 China

**Keywords:** Carbon nanotubes, Flexible application, Synergistic effect, Electrochemical sensors, Glucose sensors

## Abstract

The utilization of nanomaterials in the biosensor field has garnered substantial attention in recent years. Initially, the emphasis was on enhancing the sensor current rather than material interactions. However, carbon nanotubes (CNTs) have gained prominence in glucose sensors due to their high aspect ratio, remarkable chemical stability, and notable optical and electronic attributes. The diverse nanostructures and metal surface designs of CNTs, coupled with their exceptional physical and chemical properties, have led to diverse applications in electrochemical glucose sensor research. Substantial progress has been achieved, particularly in constructing flexible interfaces based on CNTs. This review focuses on CNT-based sensor design, manufacturing advancements, material synergy effects, and minimally invasive/noninvasive glucose monitoring devices. The review also discusses the trend toward simultaneous detection of multiple markers in glucose sensors and the pivotal role played by CNTs in this trend. Furthermore, the latest applications of CNTs in electrochemical glucose sensors are explored, accompanied by an overview of the current status, challenges, and future prospects of CNT-based sensors and their potential applications.

## Introduction

Hyperglycemia due to insulin shortage or insulin resistance is characteristic of diabetes, and is often known as diabetes mellitus [1]. Approximately 537 million individuals worldwide currently have diabetes. The number of individuals with diabetes is rising and is predicted to exceed 783 million by 2045 [2]. Glucose monitoring is a crucial component of clinical diagnostics for diabetes management [3]. Due to the high number of diabetic patients infected with COVID-19, the current coronavirus pandemic has also heightened interest in glucose control and monitoring [4]. Blood is the most common and traditional biofluid for glucose detection. However, blood collection is invasive and, therefore, uncomfortable and inconvenient for users. In recent years, advances in nanotechnology, microfluidics, and point-of-care (POC) sensing technologies, have prompted researchers to investigate alternative biofluids, such as sweat, urine, saliva, and interstitial fluid (ISF), for noninvasive, continuous, and wearable glucose monitoring [5].

Given its low cost, quick response, and user-friendliness, electrochemical analysis has attracted considerable interest in the application of glucose sensors [6, 7]. Benefiting from high selectivity, electrochemical enzymatic sensors have dominated the practical market in glucose sensing [8]. With their superior porous structure for enzyme immobilization and high conductivity, carbon nanotubes (CNTs) have received a great deal of attention in enzymatic glucose sensors [9, 10]. However, considering the low stability, pH dependence, and complex immobilization of enzymes, nanomaterials with high catalytic properties are being investigated for glucose electrooxidation [11]. This new type of glucose sensor created by these innovative technologies is known as a nonenzymatic glucose sensor.

Given their large surface area, high aspect ratio, exceptional chemical stability, and extraordinary optical and electronic properties, CNTs are also widely used in nonenzymatic sensor applications [12, 13]. Based on these properties, CNTs can be made into biocompatible and durable nanocomposites by functionalizing their surfaces with polymers, organic compounds, and biomolecules [14, 15]. In order to produce efficient and inexpensive non-enzymatic glucose sensors that are sensitive, selective and highly stable, the development of suitable or effective electrocatalysts is essential. For direct electrochemical glucose oxidation, numerous nanomaterials have been devised, including metals (Au, Pt, Pd, Ni, Cu, Co, etc.), alloys, metal oxides (NiO, CuO, Co_2_O_3_, etc.), metal sulfides, and metal–organic frameworks (MOFs) [9, 16, 17]. Notably, CNTs combined with numerous metals and metal oxides, such as Ni, Cu, and CuO, have a synergistic effect on glucose catalysis [9, 18, 19]. Moreover, with a high gauge factor and minimal hysteresis, CNTs excel in flexible devices [20]. Considering their current popularity as implantable and wearable base materials, CNTs can potentially become the focal point in the field of minimally invasive and noninvasive electrochemical glucose sensors.

**Fig. 1:**
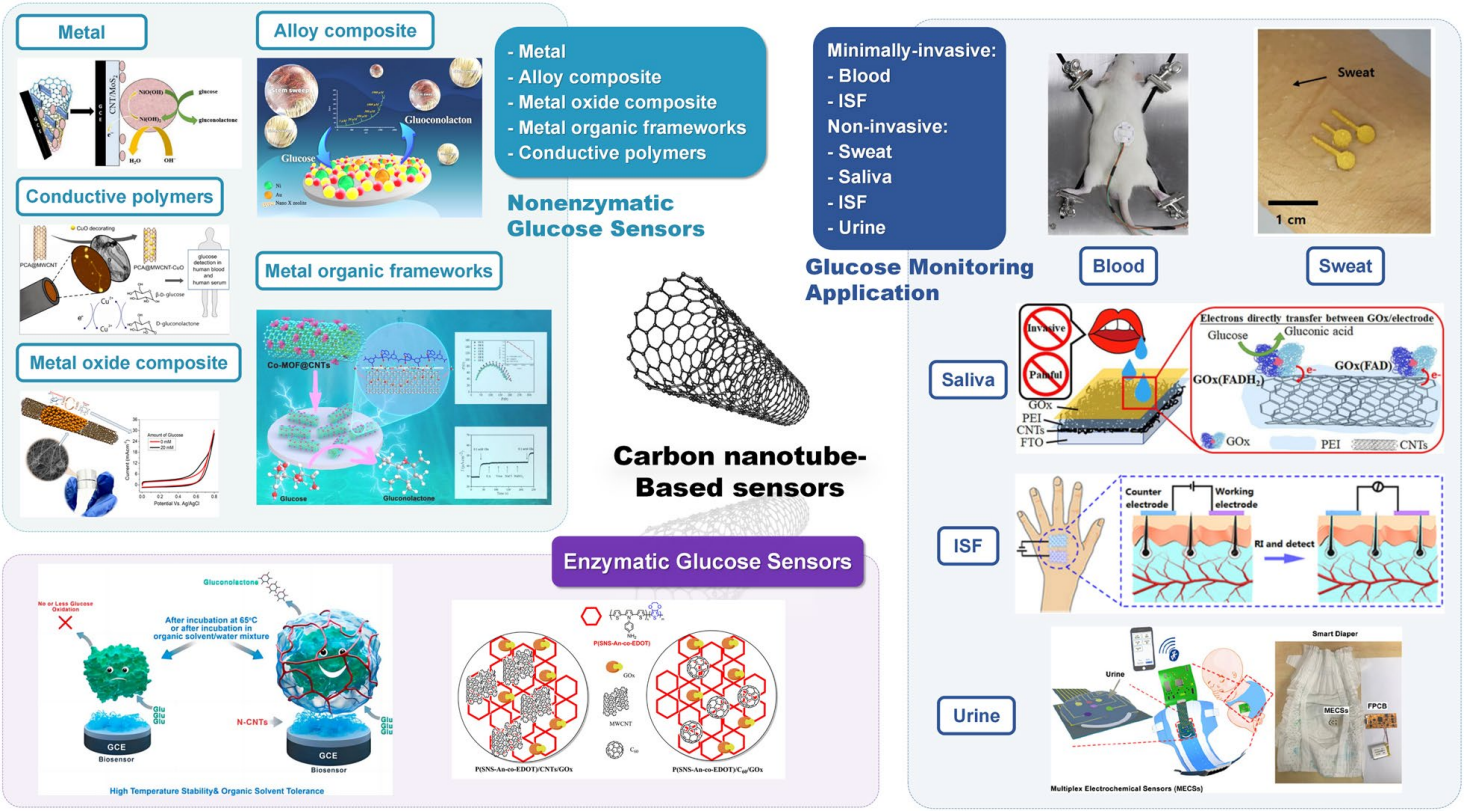
The outline of this review. The enzymatic glucose sensor (top left), the non-enzymatic glucose sensor (bottom left), and the practical application of the CNT-based glucose sensor (right)

This review aims to provide a comprehensive analysis of the current state of CNT-based glucose electrochemical sensing. Various CNT-based glucose sensors, how CNTs can be used to construct minimally or noninvasively invasive electrochemical sensing platforms, and the potential of CNT-based platforms to detect multiple diabetes biomarkers, are included in the discussion. This review examines the various biosensing mechanisms involved in CNT-based glucose sensors and the unique role of CNTs in enzymatic and nonenzymatic sensors. In conclusion, significant progress has been made in CNT-based nanomaterials for electrochemical glucose biosensing in the past five years (Fig. 1). While challenges remain, the field is poised for continued growth and innovation, with the potential to improve the lives of millions of people suffering from diabetes worldwide.

## Development of glucose sensors from enzymatic to nonenzymatic detection

**Fig. 2:**
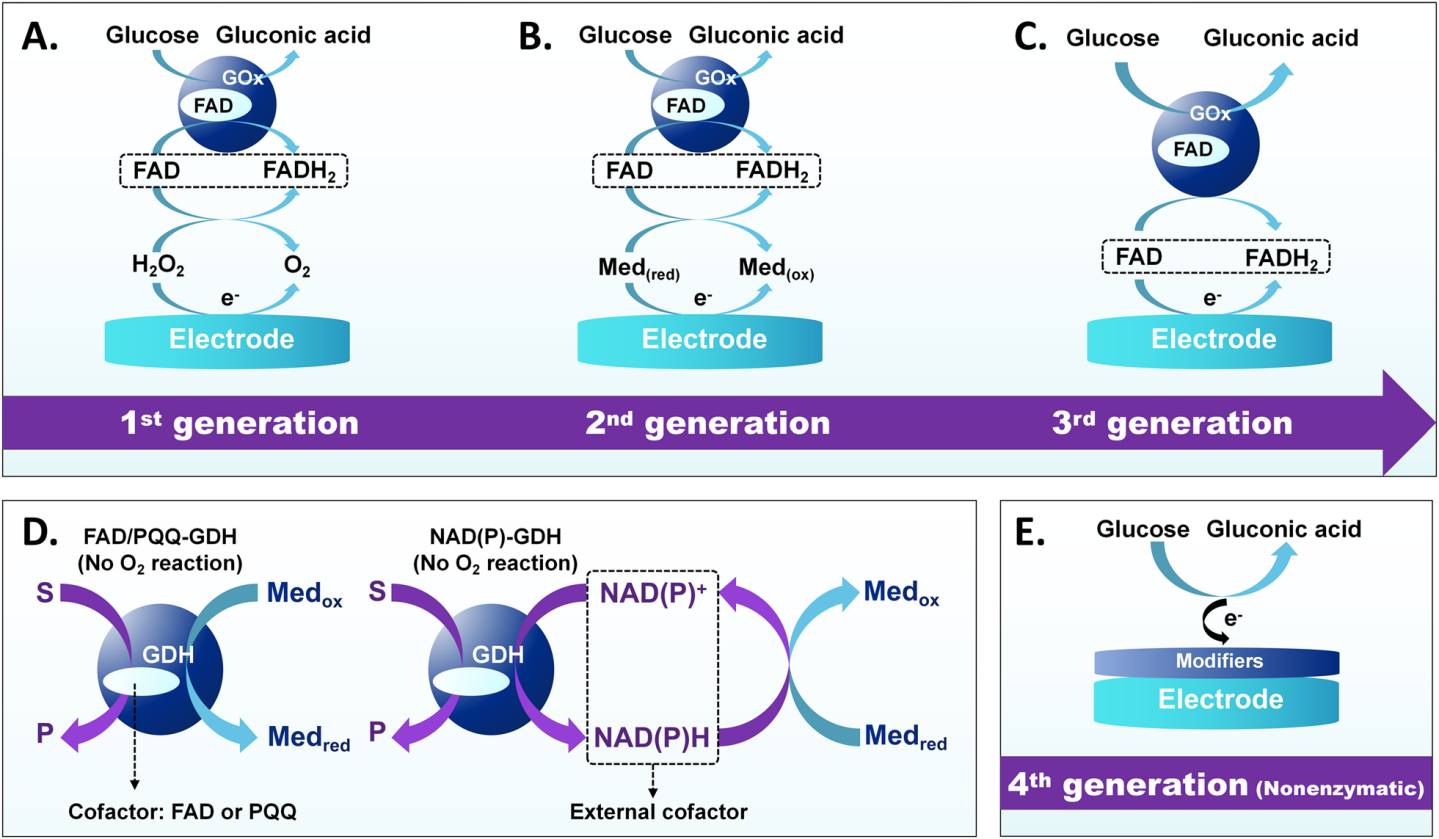
Evolution of different generations of electrochemical glucose sensors’ operating principle

The first-generation oxidase biosensors (Fig. 2A) catalyze glucose oxidation through glucose oxidase (GOx), producing gluconic acid and H_2_O_2_ [5]. The resulting H_2_O_2_ is then oxidised at the platinum electrode, with the resultant current being proportional to the glucose concentration. However, these first-generation sensors, which are reliant on oxygen and lack selectivity, require refinement [8]. To address this, the introduction of glucose dehydrogenase (GDH) for sensor preparation was suggested (Fig. 2D). GDH-based sensors function at a lower detection potential and are unaffected by the presence of oxygen in the sample [21].

Second-generation glucose sensors (Fig. 2B) employ a nonphysiological O_2_-free redox medium for electron transfer from GOx to the sensing electrode's surface [22]. These species replace oxygen and enhance electron transport. The third generation of glucose sensors eliminates the need for natural or artificial media (Fig. 2C), enabling direct electron transfer by reducing the distance between the enzyme and the electrode through direct enzyme immobilization [23].

Despite the success of enzymatic glucose sensors in addressing the problem with O_2_, the stability problems of natural enzymes still persist [24]. Nonenzymatic glucose sensors offer several advantages over enzymatic sensors, including economy, high sensitivity, and long-term stability [25]. Known as fourth-generation glucose sensors (Fig. 2E), nonenzymatic electrodes were modified with nanomaterials that can perform enzyme-like reactions to transfer electrons, especially under extreme conditions. To date, there have been numerous number of reports on nonenzymatic glucose sensors based on nanostructured metals [26], alloys [27], metal oxides [28], conductive polymers [25], MOFs [29], and other modified electrodes.

However, the performance of metal-based sensors is susceptible to absorption reaction intermediates during glucose oxidation [30]. Therefore, the choice and structure of nanomaterials greatly affect the performance of nonenzymatic sensors. To enhance the glucose catalytic efficiency, a combination of highly efficient and synergistic nanomaterials should be selected for glucose sensors.

## Principles, history, and perspective on CNTs in nonenzymatic electrochemical sensors

In 1991, CNTs were first discovered by Iijima [31]. The following year, Ebbesen and Ajayan [32] successfully obtained gram amounts of multi-walled CNTs (MWCNTs). In 1993, single-walled CNTs (SWCNTs) were independently produced by Bethune et al. [33] and Iijima and Ichihashi [34].

**Fig. 3:**
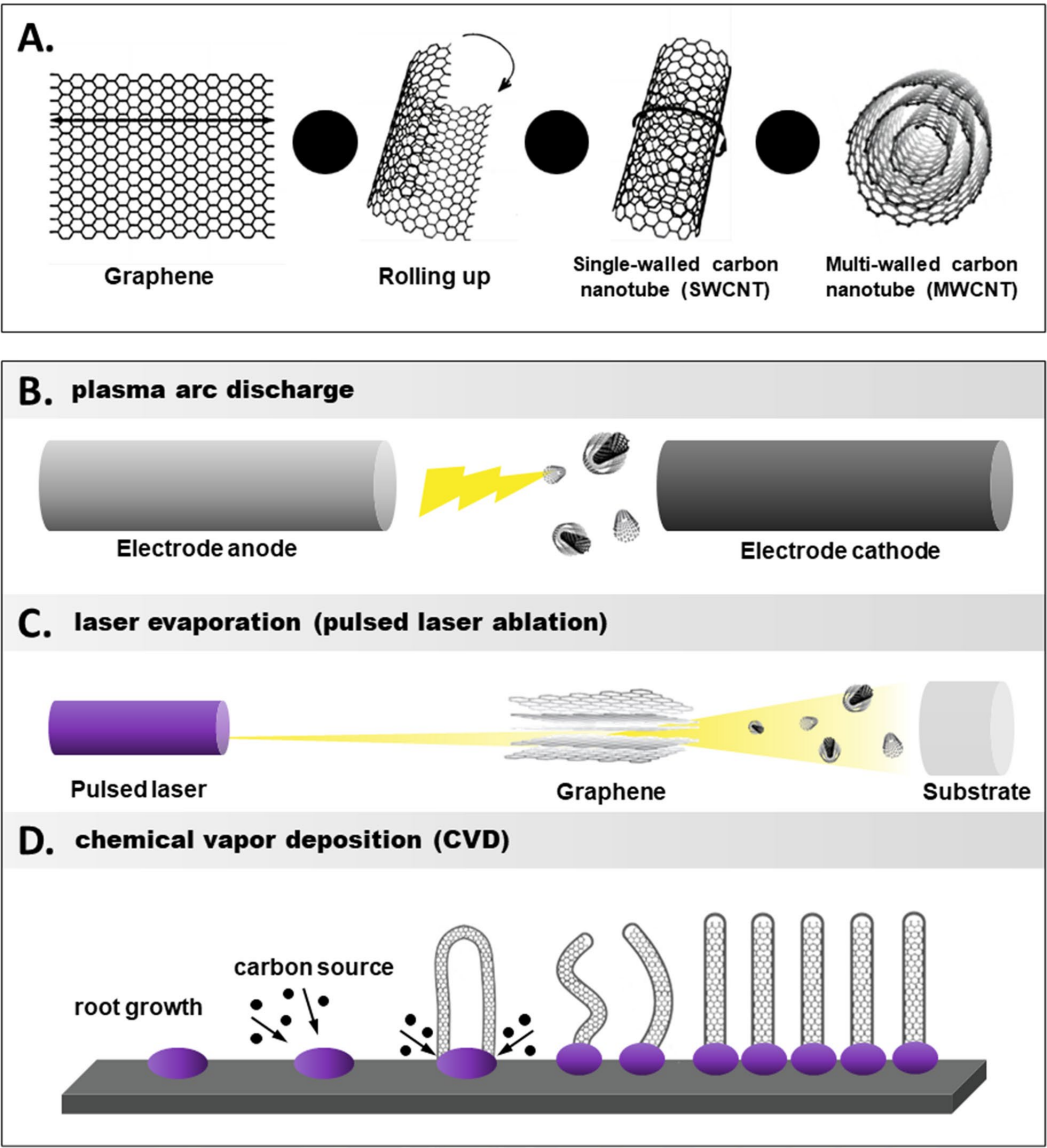
**A** Schematic structure of graphene, SWCNT and MWCNT. **B**–**D** General schemes of the plasma arc discharge method, the laser evaporation method, and the chemical vapor deposition method

In consideration of their large surface area-to-volume ratio, exceptional electrical conductivity, and biocompatibility, CNTs have been extensively investigated and utilized to manufacture electrochemical biosensors [1]. Graphene sheets can be rolled in various ways, so different types of CNTs can be produced [35] (graphene is a separate graphite layer, Fig. 3A). The curvature of graphene sheets is responsible for the electrical characteristics of CNTs. Specifically, the electron cloud of carbon transforms from having a uniform distribution in graphite along the C–C backbone to having an asymmetric distribution in and around the nanotube cylinder sheet. Owing to the distortion of the electron cloud, CNTs are electrochemically active; therefore, considerable π–electron conjugation outside the tube is formed [30]. CNTs play a dual role in electrochemical biosensors, both as carriers for immobilizing biomolecules and providing the essential electrical conductivity. The capability of the sensor is greatly dependent on the immobilization technique of the biomolecules on the CNT surface, also known as biofunctionalization. Various biofunctionalization techniques, including physical adsorption, polymer encapsulation and covalent cross-linking, have been described in studies.

CNTs can be classified into two categories based on the number of walls: single-walled and multi-walled [4, 36, 37]. Depending on the orientation of the graphene lattice around the tube axis, individual CNT walls may be metallic or semi-conductive; this property is known as chirality [38]. SWCNT films offer a unique platform for developing electronic devices because of their ability to accommodate large-area film fabrication and patterning techniques. Sensors based on flexible SWCNT films have become a hot topic for biosensor development and applications [39].

Various methods exist for producing CNTs, with arc discharge, chemical vapor deposition, and laser ablation being the most common (Fig. 2B–D). Each method has its advantages and disadvantages, as detailed in Table 1. Different synthesis methods will synthesize CNTs of different lengths and diameters. Studies have shown that CNTs with larger length-to-diameter ratios will have higher electrical conductivity [40, 41]. From Table 1, it can be inferred that CVD can synthesize CNTs with larger length-to-diameter ratio and higher electrical conductivity. As the performance of CNT-based glucose sensors is closely related to conductivity and CVD method has the potential to synthesize CNTs on a large scale, CVD may be the most promising of the current CNT synthesis methods. The future use of single-walled CNTs (SWCNTs) in electronic devices relies on obtaining pure, semiconducting SWCNTs. Commercial CNT feedstocks contain CNTs of different diameters and chirality. Techniques such as dielectric electrophoresis, density gradient ultracentrifugation, and surfactant or polar biopolymer dispersion have been developed for SWCNT separation. Conjugated polymer wrapping is a promising purification and identification strategy [42]. The strategy, with the polymer’s π-conjugated backbone interacting with the π–electron filled 2D surface of the nanotubes, thereby facilitating the break-up and dispersion of the optimal length of soluble alkyl side chains in organic solvents. The intricate structural design of the conjugated polymers allows for the selective assignment of SWCNTs with sizeable diameters or specific chirality [12].

**Table 1:** Summary and comparison of three representative methods of CNT production

Method	Process	Yield	Benefits	Drawbacks	SWCNT	MWCNT	Refs.
Arc discharge method	Under high current, carbon vaporizes and forms a hot plasma between two graphite rods spaced a few millimeters apart	< 30%	Relatively simple and inexpensive Large quantities of CNTs can be produced at once	Produce a lot of impurities substantial; purification is needed and the SWCNTs have structural defects	Short tubes with diameters of 0.6–1.4 nm	Short tubes with inner diameter of 1–3 nm and outer diameter of approximately 10 nm	[48–50]
Laser ablation	Intense laser pulses cause graphite to evaporate and form CNTs	70%	Produce high-quality, single-walled CNTs with a narrow size distribution	Expensive equipment and specialized operating knowledge Energy-intensive and may require the use of hazardous gases	Long bundles of tubes (5–20 microns), with individual diameter from 1 to 2 nm	Not very much interest in this technique, as it is too expensive, but MWCNT synthesis is possible synthesis is possible	[50, 51]
Chemical vapor deposition	Heated up to 1000 °C in an oven with/without a substrate, carbon-bearing gas such as methane decomposes on a catalyst into CNTs	95%–99%	Commercially the most developed method, easiest to scale up, good yield and quality control, high-purity SWCNTs	MWCNTs are often riddled with defects compared with SWCNTs, for which the quality is better controlled	Long tubes with diameters ranging from 0.6 to 4 nm	Long tubes with diameters ranging from 10 to 240 nm	[50, 52, 53]

Since their first application in the oxidation of dopamine in 1996 [43], CNTs have been considered one of the most promising electrode materials. Using CNTs in glucose enzymatic sensors enables electrocatalysis for glucose detection or efficient electron tunneling into the enzyme [15]. In first-generation enzyme-based glucose sensor applications, biocompatible glucose-sensing substrates comprising CNT-based composites with metal nanocatalysts have also been utilized. Using uniformly distributed Pt NPs on CNT carriers, Li’s group demonstrated 90% retention of enzyme activity after 1 month. This was attributed to the high oxygen-containing Pt-CNT density of the moiety, which facilitated enhanced biocompatibility and retention of the GOx enzyme’s bioactivity [14].

Owing to the nature of enzymes, glucose enzymatic sensors are susceptible to the effects of temperature and pH [9]. So far, various metal-, alloy-, metal oxide-, and MOF- modified CNT nonenzymatic glucose sensors have been reported [5]. For metal-based sensors, the redox reaction of metal can change glucose into gluconolactone via an electrocatalytic oxidation reaction [44]. CNTs are superior materials with a high surface-to-volume ratio and outstanding electrical conductivity, allowing for fast electron exchange between metal nanoparticles and anode surfaces [45]. Therefore, metal NPs combined with modified CNTs are particularly favored in sensors [46]. The novel MOF composites combine CNTs with functional inorganic materials, resulting in unique properties showing exceptional promise for sensor design and application [47]. In addition, the unique nanostructure and flexible properties of CNTs have demonstrated remarkable commercial potential for invasive and noninvasive glucose sensor applications.

## Recent developments in CNT-based enzymatic glucose sensors

Since Clark and Lyons completed the pioneering work on glucose biosensors in the 1960s [54], the recent development of glucose biosensors has begun to focus on enzymatic glucose sensors. Enzymatic glucose sensors have emerged as an attractive technology for noninvasive glucose monitoring, given their high sensitivity, specificity, and selectivity [55, 56]. Enzymatic glucose biosensors usually use immobilized GOx to detect glucose, in which GOx catalyzes glucose oxidation and converts it into gluconic acid [57, 58]. However, the active redox center of GOx is deeply located, making electron transfer between the enzyme and the electrode surface challenging [59]. In addition, when immobilized on the electrode surface, the GOx’s morphology may change, posing another challenge [60, 61]. Preventing the denaturation and inactivation of GOx is critical to maintaining the biosensor’s service life. Therefore, immobilizing GOx on suitable substrates is necessary to preserve its catalytic properties and bioactivity. CNT-based enzymatic glucose sensors have gained attention because of their nanoscale size and chemical, thermal, and mechanical stability, making them excellent enzyme immobilization support matrices [62, 63]. The coordination of CNT-based structures with enzymes increases enzyme loading and stability and can greatly improve the performance of sensors [64]. For instance, a modification of the screen-printed interface based on self-assembled perylene-tetracarboxylic acid/MWCNT adducts has been proposed for immobilizing enzymes efficiently [65]. The excellent electrical conductivity of CNTs also enables direct electron transfer between the enzyme and the electrode surface, leading to low operational potential.

Conducting polymers (CPs) are an essential class of functional materials that have been extensively utilized in fabricating electrochemical glucose biosensors to provide a stable, biocompatible surface for immobilizing GOx [66]. Despite their excellent electrochemical properties, CNTs can only be loaded with a limited number of enzymes when absorbed onto a surface. However, the large surface area-to-volume ratio and rich surface properties of CNTs make them suitable for chemical or physical binding to a variety of materials to form unique high-performance nanocomposites [29]. Therefore, there is a strong need to design CNT–CP nanocomposites to improve the efficiency of enzyme immobilization for electrochemical biosensing applications. Notably, the sidewalls and ends of vertically aligned CNTs (VACNTs) can provide a large surface area, resulting in fast and reliable glucose detection [67]. However, the hydrophobic nature of VACNTs makes it difficult to uniformly retain GOx molecules on the CNT surface [68]. Combining VACNTs with CPs can synergistically stabilize the biolayer on the electrode surface and form a structure suitable for glucose detection [69]. Researchers grew VACNTs on a silicon substrate using alumina as a buffer layer and iron as a catalyst through chemical vapor deposition via radio frequency sputtering and electron beam evaporation. To modify the electrode surface, they electrodeposited polyaniline (PANI) and then covalently linked it to GOx. The resulting electrodes were effective as POC glucose biosensors for detecting glucose in human plasma. The biosensors had a limit of detection (LOD) of 1.1 μM and a sensitivity of 620 μA mM^−1^ cm^−2^ over a linear range of 2–426 μM. In addition, Alhans et al. [70] conducted a comparative study on the effectiveness of SWCNT- and MWCNT-coated gold printed circuit board electrodes for glucose detection through the physical combination of attached Au-CNT. The study demonstrated that SWCNTs served as a superior sensing interface for glucose detection with the physical combination.

Polypyrrole (PPy), a common constituent of biosensors, has the advantage of facile modification and enhanced electrode selectivity and stability [71]. Huang et al. [72] fabricated acid-CNT/PPy/fluorine doped tin oxide (FTO) electrodes for non-invasive saliva glucose sensors, simply combining PPy and CNTs to achieve synergistic effects. The study used a chemical polymerisation method to deposit PPy on an FTO conductive glass substrate, which was then modified with acid-CNTs by drop-casting to fabricate acid-CNTs/PPy/FTO electrodes. However, the use of layer-by-layer CNT modification leads to a decrease in electron transfer resistance. In addition, the duration of acid treatment applied to CNTs has a significant impact on the sensing performance. In addition, PPy and polythiophene are two of the most widely used CPs because of the significant interest generated by their pyrrole and thiophene fragments [73]. However, these polymers are limited in their applications because PPy is sensitive to H_2_O_2_, and the presence of ascorbic acid and uric acid can generate H_2_O_2_ in the presence of GOx. To address these limitations, hybrid structures, such as 2,5-di(thienyl)pyrroles (SNSs), have been synthesized for biosensing applications [74]. Altun et al. [75] developed a glucose biosensor by preparing homopolymer P(SNS-An) and copolymer P(SNS-An-co-EDOT) films, doping them with carbon nanomaterials (CNTs and fullerenes) and cross-linking GOx. This innovative approach demonstrated superior biosensing interface properties compared with the previously reported P(SNS) biosensor.

**Fig. 4:**
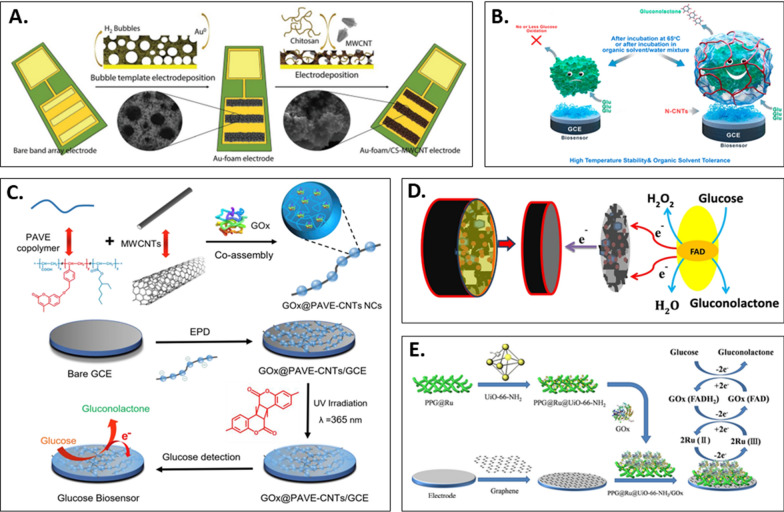
**A** Description of a 2-step electrochemical deposition process for the fabrication of Au-foam/CS-MWCNT electrodes. Reprinted with permission from Ref. [78]. **B** Illustration of SMEN’s thermal stability (65 °C) and resistance to organic solvents. Reprinted with permission from Ref. [79]. **C** Schematic illustration of GOx@ PAVE-MWCNTs NCs glucose biosensor fabrication. Reprinted with permission from Ref. [80]. **D** Glucose detection mechanism of GOx/AuNP/PANI/rGO/NH2-MWCNTs biosensor. Reprinted with permission from Ref. [84]. **E** Schematic representation of the PPG@Ru@UiO-66-NH2 sensor fabrication process and the glucose electrocatalytic reaction at the electrode interface. Reprinted with permission from Ref. [91]

A dual-enzyme system combining GOx and horseradish peroxidase (HRP) operates in a cascade mechanism, enhancing biosensor response and enzyme stability [76]. However, this system requires an oxidized mediator for oxidase regeneration [77]. To address this limitation, Juska et al. [78] presented a biosensor based on a gold ribbon array electrode with GOx and HRP. The electrodeposited gold foam increased the active surface area, enabling stable glucose detection for approximately 45 days (Fig. 4A). This sensing platform demonstrated long-term stability for glucose detection for approximately 45 days.

To ensure enzyme performance under harsh conditions, Dhanjai et al. [79] employed highly stable single-molecule enzyme nanocapsules (SMEN), rather than natural enzymes, as biometric components (Fig. 4B). The stability of the proposed SMEN-based biosensors was assessed under a range of operating conditions. Following 4 h of incubation at an elevated temperature (65 °C), the biosensor employing natural GOx lost its glucose oxidation catalytic activity. In contrast, the nGOx/N-CNT-Chi/GCE biosensor retained 56% of its initial activity. This method represents a novel and promising direction in the pursuit of robust biosensors for a multitude of applications.

Inspired by efficient molecular imprinting strategies for small molecules, Xu et al. [80] modified MWCNTs with polymeric NPs laden with enzymes to create a highly sensitive enzymatic sensing platform (Fig. 4C). The amphiphilic copolymer poly [acrylic acid-r-(7-(4-vinylbenzyloxy)-4-methyl coumarin)-r-ethylhexyl acrylate] (PAVE), which contained photocrosslinkable coumarin chain segments and carboxyl groups, was co-assembled with MWCNTs in an aqueous solution while encapsulating Gox to produce necklace-like bio-nanocomposites (GOx@PAVE-CNTs). The polymeric NPs laden with GOx were nanobeads, whereas the MWCNTs were conductive threads. The GOx@PAVE-CNT bio-nanocomposite was subsequently electrodeposited onto the electrode surface and, following photocrosslinking, formed a porous network-structured biosensing composite film. The biosensor exhibited a low LOD (0.36 μM) and ultrafast response (< 3 s) for glucose detection.

Metallic nanomaterials possess stable electrochemical properties and high catalytic activity, making them ideal candidates for maintaining biological component activity while facilitating electron transfer between proteins and electrodes. When combined with CNTs, metallic nanomaterials can exhibit improved interference resistance, sensitivity, selectivity, and stability given their compatibility with proteins [81, 82]. Zeng et al. [83] synthesized an amperometric glucose sensor through layer-by-layer assembly of MWCNTs, PANI, and AuNPs on a PTFE/GOx/AuNP/PANI/MWCNT/GCE substrate to construct a fixed GOx carrier. The synergistic effect of AuNPs, PANI, and MWCNTs reduced the molecular diffusion distance and improved the charge transfer efficiency, enabling direct electron transfer for the immobilized enzyme. Debasis et al. [84] immobilized GOx on MWCNT/PANI/graphene oxide (GO)/AuNP-functionalized SPCE to develop a highly sensitive glucose biosensor with a reduction current 13.43 times higher than that of naked SPCE and lower working potential. The glucose reaction with GOx (FAD) produced gluconolactone and GOx (FADH_2_), followed by the reaction of GOx (FADH_2_) with dissolved oxygen to form H_2_O_2_ and GOx (FAD) (Fig. 4D). The decomposition of H_2_O_2_ amplified the response, and the biosensor was validated for detecting glucose levels in human blood serum samples. To avoid oxygen use, Navaee et al. [85] designed a new platform by grafting thiamine acid and Au NPs onto amino CNT/graphene carriers in a 3D framework, followed by ultrasonic processing to arrange methionine and Au NPs as nanorods in a MOF. Electrochemical processing effectively intercepted the enzyme and facilitated subsequent electron transfer, resulting in a highly sensitive bioelectrode.

MOFs are a new category of hybrid porous materials consisting of metal ions and organic linkers with unique features including high specific surface area, size adjustable pore size, multi-functionality and high drug loading efficiency [86, 87]. MOF-based nanozymes have been extensively utilized for enzyme immobilization by using various techniques, such as absorption, covalent linkage, pore encapsulation, and coprecipitation, to preserve the enzymes’ accessibility, activity, and physical constraints [88]. To address the low conductivity of MOFs, CNTs have been introduced to enhance the electronic transmission efficiency. For instance, Song et al. [89] developed Tb@mesoMOFs on the surface of CNTs to create a Tb@mesoMOF-CNT nanocomposite, which served as a support substrate for electrochemical glucose biosensors. The use of a novel electron mediator, methylene green, and an electrocatalyst, GDH, loaded onto the surface of a GCE resulted in excellent glucose detection performance with a linear range of 25 μM to 17 mM and a LOD of 8 μM. Moreover, Dang et al. [90] prepared a mixture of metal–organic skeleton (Fe, Mn) and Au NP-anchored CNT (Au/MOFs(Fe, Mn)/CNT) by using a one-step hydrothermal method. Incorporating CNTs into graphene paper improved the conductivity, mechanical strength, and surface roughness of the flexible nanohybrid electrodes. The increased active sites of AuNPs/MOF/CNTs resulted in a enhanced peroxidase-like activity, allowing an rise on partial charge density and electron transport between the Fermi levels of MOF, Au NPs, and CNTs. Based on the cascade reaction of artificial peroxidase and GOx, glucose can be detected with increased sensitivity and specificity in the linear range of 0.005–0.3 μM with a LOD of 0.002 μM. In addition, a novel glucose sensor immobilizing GOx on a conjugated polymer and MOF composite based on ruthenium was reported [91]. A prefabricated water-soluble conjugated polymer (poly(*n*-phenylglycine)) and a MOF (UiO-66-NH_2_) were used to create PPG@Ru@UiO-66-NH_2_ via controlled chemistry synthesis (Fig. 4E). The carbonyl and amide groups on the conjugated polymer and MOF surfaces cross-linked GOx, reducing Surface carbonyl and amide groups on conjugated polymers and metal–organic frameworks (MOFs) cross-linked GOx, reducing the immobilisation potential to 0.2 V and boosting the active surface area. The PPG@Ru@UiO-66-NH_2_/GOx-coated electrode displayed a LOD of 5 μM. These MOF-based biosensors demonstrated a potential for further applications in biosensing and bioelectronics, including medical diagnostics, environmental monitoring, and food safety. A brief overview of other reported important CNT-based enzymatic glucose sensors is summarized in Table 2.

**Table 2:** CNT-based enzymatic electrochemical glucose sensors

Enzyme	Electrode	Combination with CNTs	Forms of CNT	Linear range (mM)	LOD (μM)	Long-term stability	Real sample analysis	Refs.
GOx	GOx/VACNTs/PANI	Chemical vapor deposition	VACNTs	0.002–0.426	1.1	45 days	Human serum	[69]
GOx	acid-CNTs-2.5/PPy/FTO	Layer-by-layer modification	acid-CNTs	0.01–0.7	–	14 days	Artificial saliva	[72]
GOx	GOx/P(SNS-An-CO-EDOT)/CNTs	Polymerization	MWCNTs	0.01–5.0	1.9	40 days 10% loss	Juice	[75]
GOx	GOx/HRP/MWCNTs/Au foam	Electrodeposition	MWCNTs	0.05–1.1	25	45 days	Human serum	[78]
GOx	nGOx/N-CNTs-Chi/GCE	Situ polymerization	N-CNTs	0.01–1.74	6.92	1 h 92% remained at 65 °C	–	[79]
GOx	GOx@PAVE-CNTs	Non-covalent bonding	MWCNTs	0.0001–5	0.36	35 days 92.4% remained	Human serum, Human urine	[80]
GOx	PTFE/GOx/AuNPs/PANI/MWCNTs/GCE	Layer-by-layer assembly	MWCNTs	0.0625–1.19	0.19	30 days at least 89.5% remained at 4 °C	Human serum	[83]
GOx	GOx/AuNP/PANI/rGO/NH2-MWCNTs/SPCE	Single-step pyrolysis	MWCNTs	1–10	64	30 days 96% remained at − 20 °C	Human serum	[84]
GDH	GDH/amino-CNTs/graphene/thionine/AuNPs	Cyclic voltammetry treatment	NH_2_-CNTs	0.5–6.9	50	3 weeks	–	[85]
GDH	GDH/MG − Tb@mesoMOFs-CNTs/GCE	Hydrothermal process	f-CNTs (hydroxyl group and carboxyl group)	0.025–17	8	10 days 98.2% remained	Human serum	[89]
GOx	GOx/Au/MOFs (Fe, Mn)/CNTs	Hydrothermal process	acid-CNTs	1.05–1.3 μM	0.002	–	Human serum	[90]
GOx	PPG@Ru@UiO-66-NH_2_/GOx	Electrodeposition	Not mentioned	1–10	5	7 days 95.2% remained	–	[91]

“–” means information not available in the article

*AuNP* gold nanoparticle, *CNT* carbon nanotube, *EDOT* 3,4-ethylenedioxythiophene), *f-CNT* functionalized carbon nanotube, *GCE* glassy carbon electrode, *GDH* glucose dehydrogenase, *GOx* glucose oxidase, *HRP* horseradish peroxidase, *mesoMOF* mesoporous metal–organic framework, *MG* methylene green, *MOF* metal–organic framework, *MWCNT* multi-walled carbon nanotube, *N-CNT* nitrogen-doped carbon nanotube, *NH*_*2*_*-MWCNT* amino functionalized multi-walled carbon nanotube, *PANI* polyaniline, *PAVE* poly(acrylic acid-*r*-(7-(4-vinylbenzyloxy)-4-methyl coumarin)-*r*-ethylhexyl acrylate), *PPG* photoplethysmogram, *PPy* polypyrrole, *PTFE* polytetrafluoroethylene, *rGO* reduced graphene oxide, *SNS-An* 2,5-di(thienyl)pyrrole, *SPCE* screen-printed carbon electrode, *UiO-66-NH*_*2*_ zirconium metal–organic framework, *VACNT* vertically aligned multi-walled carbon nanotube

## Recent developments in CNT-based nonenzymatic glucose sensors

To address the limitations of enzymatic sensors, such as susceptibility to temperature, pH, humidity and chemical instability, nonenzymatic sensors have emerged as a promising alternative for glucose sensing [44]. Nanomaterials have been widely used in the design of nonenzymatic glucose sensors owing to their diverse properties. In this review, we focus on CNT-based nonenzymatic glucose sensors, which utilize nanoparticles of noble and transition metals, nanostructured metal oxides, MOFs, conductive polymers, or molecularly imprinted polymers (MIPs).

### Metal and CNT-based nonenzymatic glucose sensors

#### Noble metals and CNT-based nonenzymatic glucose sensors

Noble metals, such as Au, Pt, and Pd, are commonly used in the construction of glucose sensors because of their superior electrochemical performance, stability, and repeatability. Compared with transition metals that catalyze glucose only in alkaline solution, platinum still showed excellent catalytic performance for glucose in neutral solution [92]. However, substances such as amino acids, AA, UA, and creatinine, in the presence of chloride ions, absorb intermediates on the electrode surface and interfere with the platinum-catalyzed glucose process, resulting in poor selectivity [93, 94]. To overcome these limitations, researchers have developed methods to synthesize nano-sized noble metals that increase the specific surface area and roughness. For instance, CNTs can be decorated with metal NPs to create a new type of nanohybrid material, effectively addressing the limitations of noble metals. The combination of metal Pt with CNT-based metal nanoparticle sensors has shown improved sensitivity and selectivity, and Pt-CNTs are now widely used for sensing and other applications [95]. Silva-Carrillo et al. [96] demonstrated a nanohybrid composed of Pt and MWCNTs based on 3-mercaptophenylboric acid for glucose detection. This sensor also exhibited integrated electrochemical, mechanical, and catalytic properties that were unavailable in the respective components alone.

Gold is widely recognized among noble metals for its low oxidation potential and strong selectivity in glucose oxidation [97]. It is also unique in its ability to catalyze glucose oxidation and produce H_2_O_2_ [98]. At the nanoscale, gold particles are exceptionally active and effective green catalysts, making them a prevalent research topic in the frontier between homogeneous and heterogeneous catalysis. Furthermore, based on the fact that gold nanomaterials can be easily functionalized with a wide range of organic or biological ligands, this is also the idea for constructing various combinations of gold nanocomposites in electrochemical applications [99]. Among them, Au–CNT nanocomposites are highly efficient conductive composites, and the synergistic effect of CNTs and Au makes CNTs the preferred electrochemical detection composite for Au. There are two different methods of attaching Au to CNTs: direct attachment of Au-CNTs and linked Au–(CNT nanocomposites). There are two connection types of linked Au–(CNT nanocomposites): covalent connections, such as Au–S bonds; and noncovalent connections, for instance, π–π stacking, electrostatic interactions, and hydrophobic forces. Murugan et al. [100] employed a facile strategy for constructing Au–S bonds between Au and CNT nanocomposites by using mildly oxidized MWCNTs, AuNPs, and thiol acids, including mercaptoacetic acid (MAA), mercaptopropionic acid (MPA), and mercaptosuccinic acid (MSA). The nanohybrids were coated onto a GCE, resulting in nonenzymatic glucose sensors (GC-MWCNT-MAA-AuNP, GC-MWCNT-MPA-AuNP, and GC-MWCNT-MSA-AuNP). Based on the covalent combination of Au and CNT, GC-MWCNT-MSA-AuNP electrode showed excellent electrochemical performance with a LOD of 36 nM.

Moreover, taking into account the effect of the concentration of Au solution on the composition of the MWCNT complex, Mehmood et al. [101] studied the charge transfer kinetics in the GCE modified with AuNP-MWCNT nanohybrid at varied concentrations of AuNPs in the range of 40–100 nM. The results of cyclic voltammetry and EIS demonstrated that the diffusion control, charge transfer mechanism, and concentration of AuNPs are critical factors for the charge transfer rate.

Kangkamano et al. [102] presented a novel method of fabricating a glucose sensor by modifying the mixture of MWCNT and citrate ions on a gold electrode and then freezing and thawing to easily cast MWCNTs and CS cryogel onto the electrode surface. The nonenzymatic sensor demonstrated a LOD of 0.5 µM and excellent stability, making it suitable for measuring glucose in human blood plasma. This work highlighted the great electrocatalytic and synergistic properties achieved through the combination of AuNP-decorated MWCNTs and CS cryogels, which significantly increasing the surface area.

Pd-based nanomaterials have attracted considerable interest due to their exceptional electrochemical activity and stability in the catalytic oxidation process of glucose [90]. Additionally, the ample supply of crust reserves implies reduced production costs. Synthesized through the chemical reduction of precursor Pd ions in SWCNTs-PdNPs, Pd nanoelectrodes have been discovered to display notable electrocatalytic activity for glucose oxidation, even in the presence of elevated chloride ions [103]. Ghanam et al. [104] constructed porous cauliflower-like Pd nanostructures (PdNS) on f-CNT/SPCE for glucose detection as a nonenzyme sensor suitable for neutral buffer solutions. Within this framework, F-CNTs significantly expanded the electrode's surface area, promoting the deposition of numerous Pd particles. Notably, the enhancement of the Pd catalytic activity is correlated with the morphology of the electrodeposited PdNS.

#### Transition metals and CNT-based nonenzymatic glucose sensors

Despite a large number of studies on noble metal NPs/CNTs, the large-scale application of noble metal nanoparticles is limited by the high cost and the blocking of the catalytic reaction caused by the adsorption of intermediates and chloride ions, which can be effectively addressed by non-noble metal NPs/CNTs [105]. Therefore, transition metals are widely used as electrode materials for glucose determination. Currently, the transition metals used for glucose detection, including Ni, Cu, and Co, have the advantages of easy preparation, low cost, low toxicity, adjustable surface structure characteristics, environmental friendliness, and excellent stability [106, 107].

Nickel and copper are widely used in catalytic oxidation of glucose under alkaline conditions [108, 109]. Immersing these metal (Me) electrodes in an alkaline solution produces Me (OH)_2_ species, while additional oxidation leads in the creation of MeOOH species. The catalytic components responsible for glucose oxidation are primarily Me(OH)_2_/MeOOH redox couples [110]. Therefore, such nonenzymatic sensors operate only in alkaline solutions and cannot be used to directly detect neutral biofluids, such as blood samples (which are diluted with alkaline electrolytes prior to measurement). Despite the excellent properties of transition metals, the poor dispersion of metal NPs may lead to stacking, a major factor affecting sensor performance. To solve this problem, the researchers used CNT materials as substrate, whose porous network structure expand surface area, provide abundant nucleation sites and rapid charge transfer, thus achieving efficient electrodeposition of metal NPs [111]. For instance, a microwave-assisted method was proposed to synthesize f-MWCNT supported by highly monodispersed NiNPs on GCE (Ni@f-MWCNT/GCE) [112]. According to the electrochemical results, the addition of f-MWCNTs improved the electrochemical performance of NiNPs, and the electrode showed a good linear range (up to 12 mM) and a LOD of 21 nM for glucose oxidation.

To enhance sensitivity and catalytic activity, Fall et al. [113] constructed CNT/molybdenum disulfide (MoS2) with NiNPs to construct a sensor for the rapid determination of glucose. Notably, the composite of CNT, MoS2 and NiNPs accelerates electron transfer on the electrode surface, showing a synergistic effect on conductivity. The hybrid CNT/MoS_2_/NiNP sensor showed an excellent sensitivity value of 1212 μA mM^−1^ cm^−2^ with a low LOD of 197 nM.

**Fig. 5:**
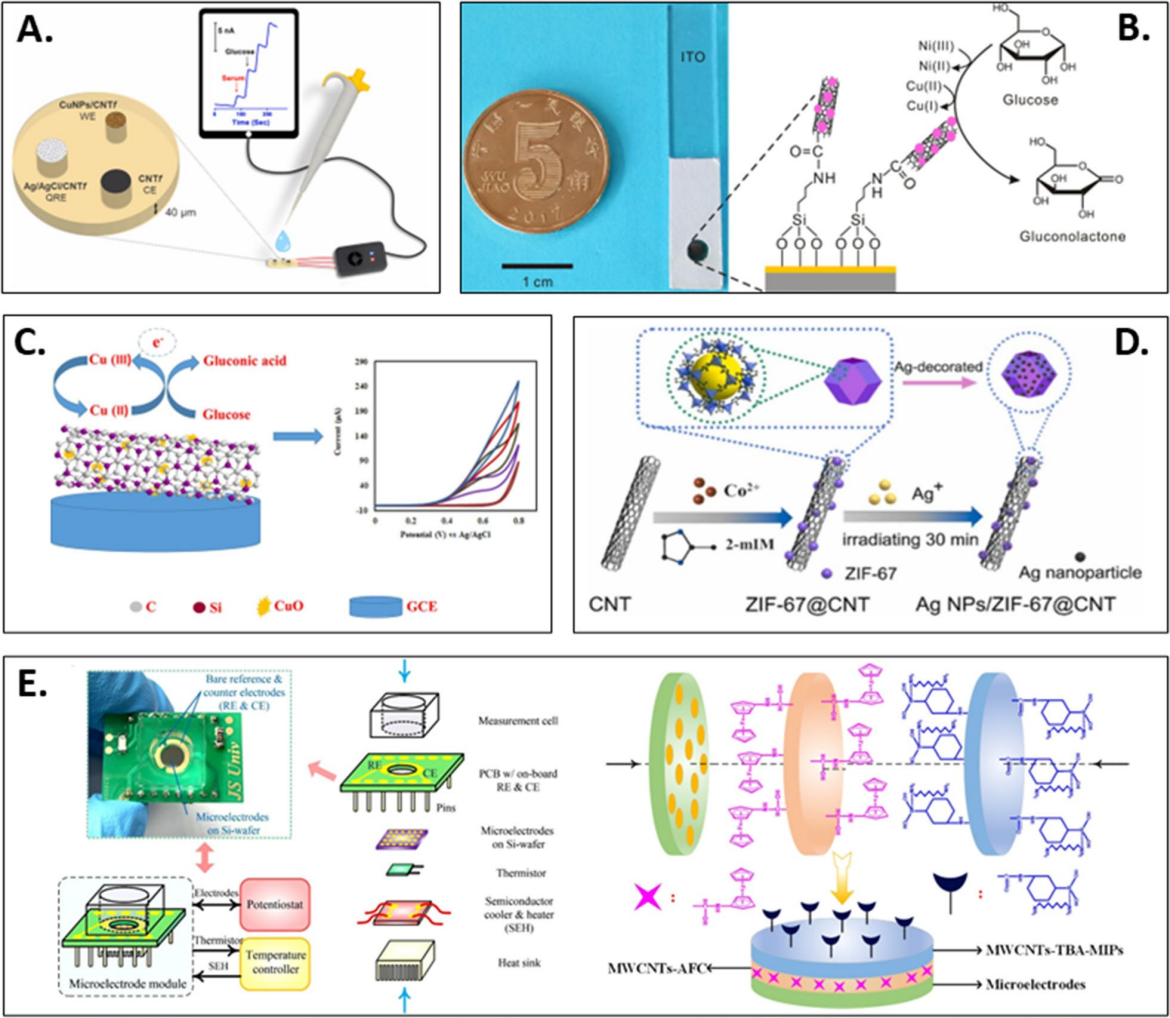
**A** Schematic illustration of CuNPs/HD-CNTf and the corresponding signals for glucose detection. Reprinted with permission from Ref. [151]. **B** Photo of the Cu/Ni-CMWCNTs sensor and the schematic depiction of the electrocatalytic reaction of glucose on the Cu/Ni-CMWCNTs composite interface. Reprinted with permission from Ref. [119]. **C** Schematic representation of the glucose oxidation reaction occurring on the Cu/g-SiCNT/CuO surface and the corresponding electrical signals. Reprinted with permission from Ref. [129]. **D** Schematic diagram of the fabrication of Ag NPs/ZIF-67 @CNT composite electrodes. Reprinted with permission from Ref. [141]. **E** Photograph, typical application, structure and detailed formation of the MWCNTs- TBA-MIPs/AFC microelectrode module. Reprinted with permission from Ref.^145^

Similar to the mechanism of Ni oxidation of glucose, the reaction of glucose catalyzed by Cu-based electrodes is highly dependent on the Cu(II)/Cu(III) REDOX pairs in alkaline media [109]. Gupta et al. [118] proposed a CNT microelectrode set consisting of three electrodes based on highly densified CNT fiber (HD-CNTf) cross sections imbedded in an inert polymer matrix with exposed open-ended CNTs at the interface (Fig. 5A). The proposed nonenzymatic CuNP/HD-CNTf microsensor has an extremely low LOD and excellent sensitivity of 1942 nAµM^−1^ cm^−2^, due to the synergistic effect of electrocatalytic CuNPs and high conductive HD-CNTf. In addition, it is worth mentioning that the microsensor catalyzes glucose independent of chloride and oxygen concentrations. Although a CNT has the unique characteristics of establishing an analysis platform and improving electron transfer, the application of a primitive CNT itself still faces challenges in case of the ease of gathering. As a result, CNTs need functionalization to be dispersed before being added to an electrochemical sensor [15]. This covalent or noncovalent functionalization has two main goals. One goal is to exfoliate nanostructures and allow them to be dispersed in an aqueous medium. The other goal is to confer special properties to the depolymerized nanostructures [5], which is more challenging. By using functionalized CNTs as a supporting material, Wang et al. [114] uniformly deposited Cu NPs on acidizing CNTs to form a composite structure of ferrocene–chitosan/CNT@Cu (Fc–CHIT/CNT@Cu) for the electrochemical glucose detection. Given the huge surface area of CNTs, the excellent electrical conductivity of Cu NPs, and the specific synergies of Fc, the electrode showed excellent glucose detection performance.

As an interesting transition metal with good catalytic activity and relatively low cost, Co can be used to electrocatalyze glucose [115]. Considering CNT as an efficient electron transfer-promoting substance, Ayranci et al. [116] used an MWCNT to embed a Keggin-type Co-polyoxometalate composed of Co(III) and Co(II) to improve electron transfer and developed an excellent nonenzymatic electrochemical glucose sensor platform. This study shows that highly conductive CNTs can not only provide high-speed paths for the transfer of ions and electrolytes but can also regulate the growth of Co.

### Alloy composite and CNT-based nonenzymatic glucose sensors

If synthesized properly, alloy electrodes with synergistic activity can provide better than single-component electrodes for glucose electrocatalysis [11]. Recently, complexes formed by bimetallic nanoparticles have emerged as a promising platform for the construction of efficient electrocatalysts [117, 118]. Combining metallic electrodes with CNT-based nanostructures can significantly improve sensitivity given the larger surface area provided by CNTs. Zhang et al. [119] prepared Cu/Ni bimetallic nanocatalysts and combined them with carboxylated MWCNTs to create different nucleation sites (Fig. 5B). The three-dimensional electron transfer network was synthesized by a concise in situ electrodeposition synthesis. Uzunoglu et al. [120] anchored PdAg metal nanoparticles on 3D MWCNT-rGO nanohybrids, combining MWCNTs and graphene to construct high performance nonenzymatic electrochemical glucose sensors.

Strong acid treatment is a common method for CNT functionalization, and Karimi-Maleh et al. [118] synthesized Pd–Ni nanoparticles supported on acid-functionalized MWCNTs (Pd–Ni@f-MWCNT) for glucose sensing. The Pd–Ni@f-MWCNT electrode demonstrated a low LOD of 26 nM, and it is suitable for the analysis of real samples due to its good reproducibility and stability.

Due to the effective work function difference between the two metals, AuPt bimetallic NPs with unique surface electron structures have significantly enhanced the efficacy of Au-induced GOx and Pt-induced catalase [121]. Wang et al. [122] prepared a new type of GCE integrated with AuPt bimetallic NPs and MWCNT mixture, which produced synergistic electrocatalytic performance in glucose sensing, demonstrating two linear ranges of 0.05–100 μM and 0.1–2.5 mM. They used GO to disperse the MWCNTs and prevent their aggregation.

Compared with bimetallic electrodes, trimetallic or multimetallic electrodes have been used in glucose sensors in recent years, likely because of the complex and difficult-to-control synthesis of these electrodes. With proper methods, most bimetallic electrodes demonstrate excellent electrochemical performance compared with multimetallic electrodes.

### Metal oxide composite and CNT-based nonenzymatic glucose sensors

Transition metal oxides have significant cost advantages over noble metals or their alloys for constructing non-enzymatic glucose sensors, such as CuO [123], NiO [124], ZnO [125], and Co_3_O_4_ [126]. Among these transition metal oxides, CuO exhibits high electrical conductivity and electrocatalytic activity, facilitating redox reactions and low overpotential [127]. Muqaddas et al. [128] used fibrous microelectrodes composed of copper oxide modified CNTs (CuO@CNTFs), which have good glucose catalytic properties and can also be used as wearable glucose sensors. The nonenzymatic glucose sensor performance was improved by a binder-free and straightforward electrodeposition method that deposited CuO nanoparticles onto the CNT fiber surface, followed by thermal treatment. The CuO@CNTFs showed a high sensitivity of 3000 µA mM^−1^ cm^−2^. The excellent performance of this microelectrode for glucose sensing is attributed to the synergistic effect of CuO nanoparticles and CNT fibers. Shakiba et al. [129] synthesized graphitized CNTs (g-SiCNT) and their Cu/CuO-based composites for electrochemical glucose detection (Fig. 5C). Compared to CNT, g-SiCNT was more beneficial as an electrocatalyst support to improve the detection sensitivity. In addition, copper was added to the g-SiCNT structure to increase the electrical conductivity. The Cu/g-SiCNT/CuO electrode showed a sensitivity of 2051 µA mM^−1^ cm^−2^.

Compared to other metal oxides, Co_3_O_4_ has poor electrical conductivity and is also limited to a low density of active sites. Combining high-conductivity materials, like CNTs, may solve this limitation [130]. Lin et al. [131] successfully constructed an electrochemical nonenzymatic glucose sensor (Co_3_O_4_-MWCNT/GCE) by synthesizing nanocomposites of Co_3_O_4_ and MWCNTs by using a one-step solvothermal method. With a LOD of 0.28 μM and a sensitivity of 2550 µA mM^−1^ cm^−2^, the new sensor can be used to monitor trace amounts of glucose in human serum samples. Han et al. [132] successfully constructed Co-Co_3_O_4_/CNT/CF nanocomposites, in which Co_3_O_4_ NPs were grown externally on CF, improving its electrical conductivity. At the same time, CNTs were grown on the surface of CF in large amounts, which further improved the electrical conductivity.

Mixed metal oxides loaded with carbon nanomaterials also show great potential in enhancing the electrochemical efficiency of glucose detection. In a recent study by Waqas [133], a new hybrid material was developed for nonenzymatic electrochemical glucose sensing. This hybrid material consisted of a nickel and Ce oxide nanocomposite supported on MWCNTs (NiCeOx/MWCNTs) and was synthesized using a one-pot hydrogen co-reduction method. This nickel oxide/MWCNT-based sensor showed excellent performance in accurately monitoring glucose in human serum samples in real time.

### MOF and CNT-based nonenzymatic glucose sensors

MOFs, also known as porous coordination polymers, have lately emerged as a novel class of crystalline porous materials owing to their complete coordination modes [134–136]. The controllable integration of MOFs and functional materials offers new multifunctional components/hybrids with superior qualities to those of the individual components [136]. By incorporating MOF particles into the pores/macropores of CNTs to develop a layered porous system, the new hybrid MOF-modified CNT system can expedite the charge transfer to boost the faradaic reactions [137], making it possible to enhance structural properties, thereby improving the adsorption and electrochemical performance, according to research [138, 139].

As a special subclass of MOF crystal, zeolitic imidazolate frameworks (ZIFs) have been a trending topic in the field of energy storage and efficient catalysts due to their porosity, large surface area, simple synthesis and long stability [38]. The integration between ZIF and CNT could vastly boost the catalytic reactions and detection performance. Qin et al. [140] obtained a Ag NP-decorated ZIF-67 functionalized CNT (Ag NP/ZIF-67@CNT) to disperse Ag NPs onto the porous ZIF-67 surface through a two-step synthetic strategy without heating. The Ag NP/ZIF-67@CNT electrode showed excellent electrocatalytic effect on glucose, with a LOD of 0.46 μM. Elizbit et al. [141] used ZIF-67 as a porous matrix to prepare an Ag@ZIF-67/MWCNT nanocomposite by encapsulating Ag NPs and decorating them with MWCNTs to add and improve the functional sites (Fig. 5D). The results demonstrated that the Ag@ZIF-67/MWCNT composite exhibited a linear range of 33–400 μM, with a LOD of 0.49 μM. The composite also showed good resistance to interference in the presence of AA and UA, suggesting promising prospects for sensing applications in vivo.

In contrast to noncovalent functionalized CNTs that rely on van der Waals interactions with the matrix, hydroxyl-functionalized CNTs can bind to the framework, mainly through hydrogen bonds. This property allows CNTs to act as crystal-growing cores, facilitating their integration with MOFs. Recently, a study demonstrated that both pristine Co-MOF and Co-MOF with hydroxyl-functionalized CNT composite can function as electrocatalytic materials for glucose oxidation [142]. Notably, the Co-MOF@CNT electrode displayed faster and more sensitive glucose detection, with a relatively lower LOD and a wider detection range than the pristine Co-MOF.

There are few experiments on the electrocatalytic properties of carbon materials derived from bimetallic MOF precursors. Therefore, Kim Se et al. [143] synthesized a hierarchical 3D nitrogen-doped CNT-anchored CoCu organic framework (NCNT MOF CoCu) by growing it directly in a high-temperature NCNT MOF CoCu-500. The NCNT MOF CoCu nanostructure had many active sites and effectively detected glucose and H_2_O_2_. The synergistic effect of bimetallic CoCu and NCNT MOF may be contributed by their unique layered nanostructures, nitrogen-doped CNT and highly graphitized carbon.

### Conductive polymer and CNT-based nonenzymatic glucose sensors

Conductive polymers (CPs) are effective dispersal materials for metal nanoparticles. The unique conjugated π-electron system in the CP structure allows for the electrochemically deposited CP to facilitate 3D growth of metal material into different shapes on the substrate, such as dendrites and lattices [144]. As a result, the resulting metal/CP nanocomposite offers higher surface area and reactant adhesion capacity than a normal spherical monometal without a CP compound, ultimately improving the electrocatalytic performance. Furthermore, a CP complexed with CNTs provides significantly greater availability of mobile electrons than composite electrode materials that have not been modified with CP. The inclusion of CPs in sensors allows them to adapt to physiological pH conditions and varying temperatures, enhancing their potential for applications [145].

A novel nonenzymatic electrochemical glucose sensor was successfully fabricated utilizing pencil graphite electrodes (PGEs) modified with suspensions of poly(3,4-ethylenedioxythiophene): poly(styrenesulfonate) (PEDOT: PSS) and MWCNTs, accompanied by CuO NPs [146]. The employment of PGEs as a substrate for nonenzymatic glucose electrodes constitutes a divergence from the conventional use of PGEs in enzymatic glucose biosensors. The modifier suspension, comprising PEDOT:PSS and MWCNTs, was integrated to augment electron transport within the sensor. The inclusion of MWCNTs led to further improvements in the current response. This effect can be ascribed to the ability of MWCNTs to enable rapid electron transport kinetics during glucose oxidation and to their contribution of an expansive surface area to volume ratio. Through appropriate suspension modification, PGEs have revealed potential for nonenzymatic glucose biosensors, potentially paving the way for innovations in glucose sensing technology.

Previous investigations have shown that the chemical polymerization of caffeic acid (PCA) on MWCNTs can enhance the electrocatalytic characteristics and sensing performance of NADH. This amplification is attributed to the formation of quinone/hydroquinone redox couples [147]. Consequently, Kuznowicz et al. [148] devised a novel nonenzymatic glucose sensor based on PCA@MWCNTs adorned with CuO NPs (PCA@MWCNT-CuO), boasting a sensitivity of 2412 μA mM^−1^ cm^−2^. The synergistic interplay of PCA, MWCNTs, and CuO likely accounted for the outstanding properties observed in glucose detection. These results validate the detection capability of nonenzymatic sensors for glucose in human serum and blood samples.

As an artificially constructed polymeric receptors, MIP can mimic the natural receptors in molecular recognition properties [149]. The molecular imprinting method to produce MIPs includes the polymerization and subsequent removal of functional monomers in the presence of a template, leaving corresponding holes in the polymer matrix. A MIP of glucose can be efficient for the precise detection of biological samples in consideration of its ease of attaining high binding affinity with glucose [150]. Using a teamed boronate affinity-based molecular imprinting, Xu et al. [145] prepared a temperature-regulative module, microelectrode module (MEM), which can detect the blood detection level in a physiological environment (Fig. 5E). The researchers also developed MWCNT-TBA-MIP/AFC MEMs by filling the measurement cell with ferrocene-modified MWCNTs (MWCNTs-AFCs) and borate affinity-based MIP-modified MWCNTs (MWCNTs-TBAMIPs). Under physiological conditions, the MWCNT-TBA-MIP/AFC MEM detected glucose with a low LOD (0.61 μM). The application of the MWCNT-TBA-MIP/AFC MEM in the selective and accurate measurement of glucose in human serum samples, revealed its viability for further application**.** Table 3 briefly describes other essential CNT-based non-enzymatic glucose sensors reported.

**Table 3:** CNT-based nonenzymatic electrochemical glucose sensors

Classification	Electrode	Synergistic effect with CNT	Detection method	Linear range (mM)	LOD (µΜ)	Electrolyte	Detection sensitivity (µA mM^−1^ cm^−2^)	Inferences	Stable days	Real sample analysis	Refs.
Noble metals and CNT-based	3MPBA-Pt NPs/CNTs	Not mentioned	CV	0–10	4.5	0.5 M KOH	22.25 mVmM^−1^	UA, AA, Fru, Ur, acetaminophen	–	Human urine	[96]
	GCE-MWCNTs-MSA-AuNPs	Not mentioned	Amperometry	0.12–3.5 µM	0.036	KOH	22.9 µA cm^−2^	UA, H_2_O_2_, IM drug	–	Urine	[100]
	AuNPs-MWCNTs-CS cryogel/AuE	Yes	Amperometry	0.001–1.0	0.5	0.050 M NaOH	3.50 ± 0.06 µA mM^−1^	AA, UA, DA and Cl^−^	90%, 525 inj	Human plasma	[102]
	PdNS/f-CNT/SPCE	Yes	Amperometry	1–41	95	0.1 M PBS (pH = 7.4)	9.3	AA, DA, Gal, PCM	–	Human blood	[104]
	Ni@f-MWCNT/GCE	Not mentioned	CV and CA	0.025–1	0.021	0.1 M NaOH	70,000	AA, DA, UA, NaCl	> 10 weeks	Human blood serum	[112]
	CuNPs/HD-CNTf	Yes	CV and Amperometry	%1.%2–1	28 nM	0.1 M NaOH	1.942	AA, DA, UA, NaCl, Fru, Lac, Suc	7	Human serum, human urine	[151]
	Fc-CHIT/CNT@Cu	Yes	CV and CA	0.2–22	13.52	0.1 M NaOH	1.256	UA, AA, DA	22	–	[114]
	Co-POM/MWCNT	Yes	CV and Amperometry	0.1–10.0	1.21	0.1 M NaOH	256.4	AA, PH	5 weeks	Coke, juice	[116]
Alloy and CNT- based	Pd–Ni@f-MWCNT/GCE	Not mentioned	CV and CA	Up to 1.4	0.026	0.1 M NaOH	71	AA, DA, UA, Lac, Fru, NaCI	10 weeks	Human blood serum	[118]
	GO/MWCNT/Au@Pt/GCE	Yes	DPV	0.00005–0.1 and 0.1–2.5	0.042	0.1 M NaOH	330	AA, DA, L-GSH	–	Human serum	[122]
Metal oxide composite and CNT- based	CuO@CNTFs	Yes	CA	up to 13	1.4	0.1 M KOH	3000	–	–	–	[128]
	Cu/g-SiCNT/CuO	Yes	CV and Amperometry	0.001–4.48	0.8	0.1 M KOH	2051	DA, UA, AA	2 weeks, 95.5%	Human blood plasma	[129]
	Co3O4-MCNT / GCE	Not mentioned	Amperometry	0.001–0.122	0.28	0.1 M NaOH	2550	DA, AA, K+, Na^+^, Mg^2+^, Ca^2+^	2 weeks	Human serum	[131]
	Co-Co3O4/CNT/CF	Not mentioned	Amperometry	0.0012–2.29	0.4	0.1 M NaOH	637.5	UA, DA, KCl, Gal	16	–	[132]
	NiCeOx/MWCNTs	Yes	CV and CA	0.007–0.466, 0.466–3.44	1.8	0.1 M NaOH	271.53 and 429.95	l-Arginine, AA, UA, DA, H_2_O_2_, NaCl	7	Human serum	[133]
MOF and CNT-based	Ag NPs/ZIF-67 @CNT-1.0/GCE	Yes	CV and Amperometry	0.010–7.0	0.46	0.1 M NaOH	469.4	UA, DA, AA, Arg, Cys	28	–	[140]
	Ag@ZIF-67/MWCNTs/NF	Not mentioned	CV and Amperometry	0.033–0.4	0.49	0.1 M NaOH	13.014	UA, AA	–	–	[141]
	Co-MOF@CNTs	Yes	CV and Amperometry	0.01–0.06	21	0.1 M NaOH	104.37	UA, Ur, inorganic salt	–	–	[142]
	NCNT MOF CoCu	Not mentioned	CV	0.05–2.5	0.15	0.1 M PBS (pH = 7.0)	1027	UA, DA, AA	60, 96%	Human serum	[143]
Conductive polymer and CNT- based	PEDOT: PSS-CuO-MWCNTs/PGE	Not mentioned	CV and CA	Up to 10	0.23	0.1 M NaOH	663.2	UA, DA, AA, Fru, Suc, Lac	30, 93%	–	[146]
	GC/PCA@MWCNT-CuO	Yes	CV and Amperometry	0.002–9	2.3	PBS (pH = 7.4)	2412	UA, AA, l-cyst, DA	–	Human serum, human blood	[148]
	MWCNTs-TBA-MIPs/AFC MEM	Yes	CV and DPV	0.001–0.18	0.61	10 mM PBS (pH = 7.4)	–	UA, DA, AA, GA, Xyl	–	Human serum	[145]

“–” means information not available in the article

*CNT* carbon nanotube, *MWCNT* multi-walled carbon nanotube, *3MPBA* 3-mercaptophenylboronic acid, *AA* ascorbic acid, *AFC* aminoferrocene, *AuE* Au (Gold) electrode, *Arg* arginine, *CA* chronoamperometry, *CF* carbon foam, *Co-POM* K7[CoIIICoII(H2O)W11O39]⋅15H_2_O, *CS* chitosan, *Cys* cysteine, *CV* cyclic voltammetry, *Amp* amperometry, *CNT* carbon nanotube, *CNTF* carbon nanotube non-woven fabrics, *DA* dopamine, *DPV* differential pulse voltammetry, *Fc-CHIT* ferrocene-chitosan, *f-CNT* functionalized carbon nanotube, *Fru* fructose, *FTO* fluorinetin oxide, *GA* glucoamylase, *Gal* galactose, *GC* glassy carbon, *GCE* glassy carbon electrode, *GO* graphene oxide, *g-SiCNT* graphenic SiC nanotube, *HD-CNTf* highly densified carbon nanotube fiber, *IM* imatinib mesylate, *LA* lactic acid, *Lac* lactose, *Fru* fructose, *Suc* sucrose, *l-GSH*: l-Glutatione reduced, *MCNT* multiwalled carbon nanotube, *MEM* microelectrode module, *MOF* metal–organic framework, *l-cyst* l-cysteine, *MSA* mercaptosuccinic acid, *MWCNT* multi-walled carbon nanotube, *MWCNTs-TBA-MIP* multi-walled carbon nanotube-teamed boronate affinity-based molecular imprinting polymer, *NCNT* nitrogen-doped carbon nanotube, *NF* nickel foam, *NiCeOx* nickel and cerium mixed oxide, *NP* nanoparticle, *PBS* phosphate buffer, *PCA* poly caffeic acid, *PCM* paracetamol, *PdNS* Pd nanosheets, *PH* pohenol, *PEDOT:PSS* poly(3,4-ethylenedioxythiophene):poly(styrenesulfonate),* SPCE* screen-printed carbon electrode, *UA* uric acid, *Ur* urea, *Xyl* xylose, *ZIF-67* co-based zeolitic imidazolate framework

## Application of CNTs in electronic devices for glucose monitoring

So far, the excellent electrical conductivity and morphological plasticity of CNTs have shown exemplary performance in various electronic devices [152–154]. CNT -based electrochemical sensor applications for glucose detection are classified into minimally invasive and noninvasive sensors. In the former, CNTs are applied as flexible implant carriers, while in the latter, CNTs can be used as flexible base films or be doped in various modified conducting polymers. The use of CNTs has significantly enhanced the physical characteristics and electrical conductivity of electrodes, creating a new market force for glucose-detecting materials. CNTs are also an excellent carrier of multiple markers, in line with the development of solid glucose sensors for multichannel simultaneous detection. In addition to showing the functional characteristics of CNTs, we also list the specific analytes of multiple markers in Table 4.

**Table 4:** Detecting glucose in body fluid by minimally invasive and non-invasive CNT-based electrochemical glucose sensors

Real sample analysis	Recognition component	Analyte (s) detected simultaneously in the same system	Linear range (mM)	LOD (μM)	Detection sensitivity (μA mM^−1^ cm^−2^)	Interference	Detection method	Stable days	Refs.
Blood	Glucose SSF	H_2_O_2_, Ca^2+^, K^+^, Na^+^, H^+^	2.5–7.0	50	5.6 nA μM^−1^	Mal, Man, Lac, NAD^+^, AA	CA	28	[156]
Blood	GOD MPs/CNT@GHM	–	0–24	–	25 nA mM^−1^	Lac, UA, AA, AP	Amperometry	9	[158]
ISF	GDH/pMB/Au-MWCNTs/AuELOX/ pMB/Au-MWCNTs/AuE	Lactate	0.05–5 and 0.01–0.1	7	405.2, 797.4	AA, UA, LA	CA	30	[160]
Sweat	GOx/HRP/CNT-EVA	–	up to 1.0	3	270 ± 10	NaCl, Ur, LA	Amperometry	7	[169]
Sweat	CoWO4/CNT/CNT − AuNS	pH	up to 0.3	1.3	10.89	AA, UA, Ur, AP	CA	10	[170]
Sweat	CNTs/Ti3C2Tx/PB/CFMs	Lactate, pH	0.01 to 1.5	0.33	35.3	AA, UA, LA	CA	15	[173]
Sweat	GOx/Chitosan/CNT ink-PB/CNFs	Lactate	0.025–3	25	–	AA, UA, Cr	Amperometry	28	[174]
ISF	G/CNTs/GOx composite textile	–	0.12–3.5 μM	0.06	14.45 ± 2.97	UA, AA, LA, KCl, NaCl	Amperometry	30	[177]
Saliva	FTO-CNTs/PEI/GOx	-	0.07–0.7	70	63.38	AA, UA, DA	CV	14	[178]
Saliva	GCE-SWCNT/rGO/CoPc	–	0.0003–0.50 and 0.50–5.0	0.12	992.4	Suc, AA, UA, Fru, DA, K^+^, Na^+^, Cl^−^, Ur	CA	40	[179]
Urine	MECSs	Na^+^, K^+^, H_2_O_2_, UA	2.8–5.6	15.5	2.71 μA mM^−1^	Ur, KCl, NaCl	CA	6	[182]
Urine	PtAu/CNTs nanozyme	pH	0.9–40	400	–	AA, UA, Gal, Fru, Na^+^, K^+^, Ca^2+^, Cr, Ur	Potentiometry	60, 7 in human urine	[181]

“–” means information not available in the article

*CNT* carbon nanotube, *MWCNT* multi-walled carbon nanotube, *ISF* interstitial fluid, *GCE* glassy carbon electrode, *GOx* glucose oxidase, *SSFs* single-ply sensing fibres, *GHM* gradient-structured hollow fiber membrane, *pMB*: polymethylene blue, *LOX* lactate oxidase, *GDH* glucose dehydrogenase, *HRP* horseradish peroxidase, *EVA* ethylene–vinyl acetate copolymer, *AuNS* Au nanosheets, *G* graphene, *FTO* fluorine-doped tin oxide, *PEI* polyethylenimine, *SWCNT* single-walled carbon nanotube, *rGO* reduced graphene oxide, *CoPc* cobalt phthalocyanines nanohybrid, *MECSs* multiplex electrochemical sensors, *AA* ascorbic acid, *AP* acetaminophen, *CV* cyclic voltammetry, *DA* dopamine, *Fru* fructose, *Gal* galactose, *LA* lactic acid, *Lac* lactose, *Mal* maltose, *Suc* sucrose, *Mal* maltose, *Man* mannose, *rGO* reduced graphene oxide, *Ur* urea, *UA* uric acid, *Cr* creatinine, *CA* chronoamperometry

### Minimally invasive CNT-based glucose-monitoring biosensors

The development of functional implantable biosensors for continuous glucose monitoring is essential for optimal insulin therapy in diabetic patients. However, challenges such as inflammation, biological incompatibility, and immunological responses often limit the in vivo function of enzymatic electrochemical sensors. To address these issues, the size and invasiveness of implantable sensors need to be minimized to reduce confounding variables. The mechanical mismatch between the implant and biological tissue can also lead to incorrect readings and long-term tissue damage, making flexible implantation a complex issue [155].

To tackle these challenges, Wang et al. [156] introduced a solution by utilizing functionalized MWCNTs twisted into helical fiber bundles that mimic the layered structure of muscles. These high-strength CNT fibers establish a stable interface between tissue and fiber, demonstrating excellent biointegration. A glucose-responsive GOx layer immobilized on chitosan-coated fibers further enhances sensor functionality. To improve sensor stability and selectivity, micropores were introduced using Nafion and glutaraldehyde. These glucose-sensing fibers operate effectively within the range of 2.5–7.0 mM, which is suitable for detecting glucose in bodily fluids. Importantly, this device enables long-term monitoring of biomolecules in vivo with spatial resolution and real-time feedback. In an experiment, glucose levels in a cat’s venous blood were successfully monitored for 28 days. This multifunctional CNT spiral fiber bundle holds promise as a versatile implantation strategy for various biomedical and healthcare sensing applications.

**Fig. 6:**
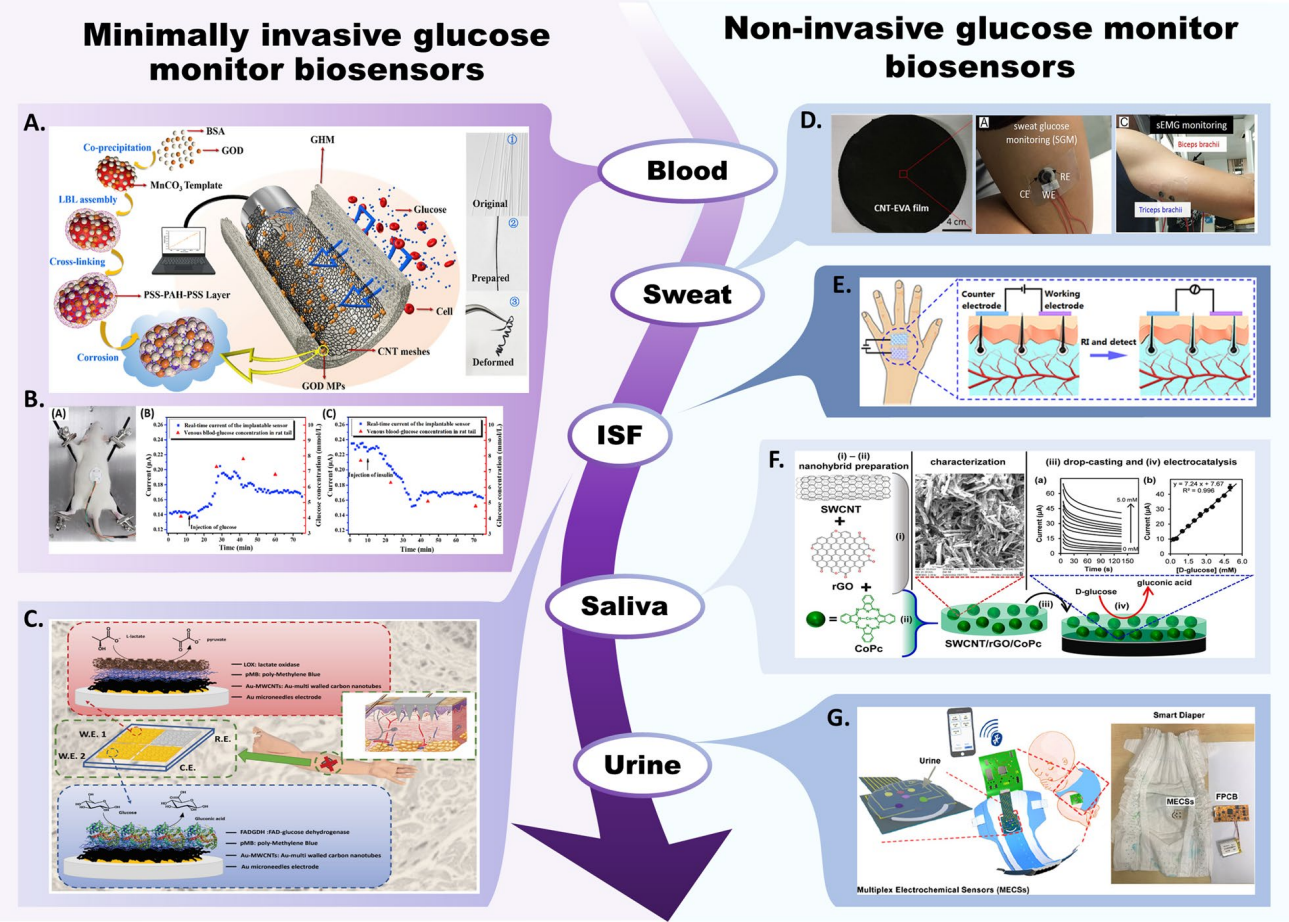
**A** Schematic representation of the preparation of GOD MPs (left) and the working electrode of the biosensor (center); photographs of the original GHM, the prepared enzyme electrode and the deformed electrode (right). **B** In-vivo test in rats. An image of the glucose biosensor attached to a rat's skin (left); current responses from the implanted sensor following glucose (middle) and insulin injections (right). Reprinted with permission from Ref. [158]. **C** Schematic diagram of a microneedle-based biosensor array for simultaneous monitoring of lactate and glucose in artificial interstitial fluid. Reprinted with permission from Ref. [160]. **D** Photo of wearable biosensors for monitoring sweat glucose and surface electromyography. Reprinted with permission from Ref. [169]. **E** Schematic diagram of a non-invasive blood glucose sensor using two textile-like electrodes (left) and ISF extraction through the reverse iontophoresis (RI) process (right). Reprinted with permission from Ref. [177]. **F** Schematic representation of the SWCNT/rGO/CoP preparation (left), characterizations (middle) and electrocatalysis processes and signals generated (right). Reprinted with permission from Ref. [177]. **G** Photo of the smart diaper and schematic diagram of the working process. Reprinted with permission from Ref. [182]

Despite these advances, challenges remain, including the deactivation and leakage of GOx, leading to reduced stability and limited sensor longevity [157]. Addressing this, Huang et al. [158] introduced a molecular sieve platform to enhance electrode efficiency in the physiological environment. They employed a gradient-structured hollow fiber membrane based on MWCNTs coimmobilized with GOx microparticles. This membrane demonstrated effective filtering of substances such as red blood cells, enhancing glucose detection accuracy (Fig. 6A). CNT meshes played a crucial role in catalyzing electron formation and transmission from enzymes to the working electrode during glucose monitoring. In vitro tests on rats indicated excellent sensing linearity within the range of 0 to 24 mM (Fig. 6B).

Implanted enzymatic continuous glucose monitors also face challenges related to poor linearity and a narrow sensing range due to the hypoxic interstitial fluid environment [159]. Bollella et al. [160] introduced a pain-free microneedle-based biosensor array capable of synchronous monitoring of lactate and glucose in the interstitial fluid (ISF) (Fig. 6C). They modified the gold surface of microneedles through electrodeposition of Au MWCNTs, followed by electropolymerization of the redox mediator methylene blue. The synergy of the AuMWCNT/polyMB platform with lactate oxidase and FAD GDH enzymes enabled continuous monitoring of lactate and glucose levels in the artificial ISF. The glucose biosensor demonstrated a sensitivity of 405.2 ± 24.1 μA mM^−1^ cm^−2^ and a limit of detection of 7 μM.

### Noninvasive CNT-based glucose-monitoring biosensors

In recent years, significant progress has been made in developing noninvasive systems using electronic devices on flexible substrates or directly applied to the skin [161]. These systems often target body fluids such as saliva, sweat, ISF, and urine. CNTs, with their high electroconductivity, offer promising possibilities for integration with various metals and polymers, enhancing noninvasive glucose monitoring. CNTs play a crucial role in improving flexibility and conductivity in wearable applications. For instance, MWCNTs can form bridges with silver nanowires in a hybrid network, preventing fracture under bending strain [162]. Highly stretchable conductive CNTs and polyurethane nanofiber spiral yarns have also been developed, demonstrating stable conductivity and recovery during deformation [163]. The firm winding of CNTs was utilized to form a more stable conductive network, resulting in a yarn with stable conductivity and recovery in the 900% deformation range, and maintaining conductivity when stretched to 1700%. Buckypaper, a CNT-based paper-like membrane, has shown potential for flexible and wearable electrochemical devices, contributing to innovative medical device and wearable technology development [164]. This holds significant implications for the development of innovative technologies in the field of medical devices and wearables.

Human sweat, containing valuable biomarkers, holds promise for noninvasive health status monitoring [165]. Glucose can diffuse from blood into sweat, establishing a connection between blood glucose and sweat glucose levels [166]. Compared to other body fluids, such as blood, ISF, and urine, sweat glucose can be more conveniently detected by biosensors [167, 168]. Xia et al. [169] introduced a mediator-free wearable biosensor for real-time glucose sensing in sweat (Fig. 6D). They developed a flexible and hierarchical meso/macroporous film comprising CNTs and ethylene–vinyl acetate (EVA) copolymer as the sensing substrate. The film's 3D conductive nanoporous structure enabled direct electron transfer-based electrocatalysis, eliminating the need for a mediator in glucose monitoring. The CNT-EVA film was functionalized with a GOx-HRP bienzyme, resulting in biosensors with exceptional selectivity and high sensitivity (270 ± 10 μA mM^−1^ cm^−2^).

Based on the stretchable nonenzymatic AuNS/CNT electrode, Oh et al. [170] created a wearable electrochemical biosensor based on a stretchable nonenzymatic AuNS/CNT electrode to detect glucose and pH in sweat. CoWO_4_ NPs were immobilized on CNTs with a large surface area, enabling selective glucose detection without interference from other chemical components and ions in sweat. This biosensor exhibited long-term stability over 10 days without a significant decrease in sensitivity.

With the study of MXene nanomaterials emerging as a focal point of interest, MXenes exhibit unparalleled potential for application in glucose biosensors. Glucose biosensors displaying high sensitivity, extensive detection range, excellent thermal stability, and dependable selectivity are now in development [171]. As a representative member of the MXene family, Ti_3_C_2_T_x_ MXene, known for its metallic conductivity, manifests remarkable electrochemical activity and supports the immobilization of biomolecules, advantages that are conducive to creating sensors for various diagnostic applications [172]. Leveraging Ti_3_C_2_T_x_ MXene nanocomposites, Lei et al. [173] engineered a stretchable, wearable, and modular multifunctional biosensor. This device, incorporating a novel Ti_3_C_2_T_x_/Prussian blue (Ti_3_C_2_T_x_/PB) composite, was designed for the simultaneous monitoring of sweat glucose, lactate, and pH. Recognizing that the limited oxygen supply in the enzymatic reaction zone could restrict the upper detection limit, linearity, sensitivity, and accuracy according to Fick’s law, the team designed biosensors with open-air pores. These pores form a solid–liquid–air three-phase interface, allowing a consistent oxygen supply and achieving a higher sensitivity of 35.3 μA mM^−1^ cm^−2^ for glucose detection. Furthermore, the simultaneous monitoring of pH values enhances the accuracy of the sensors by adjusting glucose and lactate concentrations through the calibration plot for different pH values. During electrode fabrication, the Ti_3_C_2_T_x_/PB hybrid nanosheets intermingled with each other, and CNTs became entangled, resulting in nested structures within the restacked Ti_3_C_2_T_x_/PB layers. Interestingly, the combination of CNTs and CaCO_3_ particles, followed by dissolution in hydrochloric acid, produced a porous and ultrathin film, enhancing oxygen transport. The ensuing intercalation and winding of CNTs through the layers yielded larger surface-active sites, aiding enzyme immobilization. Impressively, the glucose sensor demonstrated consistent stability, with negligible current fluctuation over 15 days without additional calibrations.

Nadtinan Promphet et al. [174] reported on a wristwatch sensor designed for the real-time, simultaneous detection of glucose and lactate in sweat. The cotton thread electrode was modified with cellulose nanofibers, CNT ink, PB, and chitosan to enhance liquid adsorption, bioreceptor immobilization, and sensor performance while concurrently minimizing skin irritation. In this study, the use of water-based CNT ink facilitated a more straightforward coating process due to its low viscosity, which led to more profound penetration into the cotton thread-based working electrode. PB was added to the CNT ink to further enhance the electrocatalytic properties of the thread-based electrode. The wristwatch sensing device offers a linear range of 0.025–3 × 10^–3^ m with a detection limit of 0.025 × 10^−3^ m for glucose, a phenomenon that can likely be ascribed to the remarkable synergy between GOx and CNT ink-PB-modified conductive thread-based electrodes. Notably, the current responses for glucose and lactate remained above 80% after 28 days, possibly due to the chitosan membrane on the thread-based electrode, which aids in preserving the enzymatic activity of both enzymes.

ISF-based noninvasive blood glucose sensors typically require the construction of at least three or more electrodes, complicating the device design [175, 176]. To streamline the fabrication process, Yao et al. [177] devised a wearable noninvasive glucose sensor utilizing a G/CNT/GOx composite textile for the working electrode and a G/CNT/Ag/AgCl composite textile for the counter electrode (Fig. 6E). The CNTs acted as efficient conducting platforms for GOx, marking the first integration of ISF extraction and blood glucose monitoring modules into a unified device, resulting in semicontinuous blood glucose observation. The textile-like electrodes confer these sensors with great flexibility and wearability, thereby permitting integration with other electronic components for comprehensive human health management and monitoring.

The advancement of saliva testing heralds an era of noninvasive and pain-free glucose assessment. Lin et al. [178] engineered a novel electrode for saliva-based, noninvasive glucose sensing. CNTs were cultivated via chemical vapor deposition on a glass substrate coated with FTO, followed by GO immobilization using electrostatic force and polyethylenimine (PEI). This study revealed that the CNT forest substantially bolstered charge transfer, and the networked CNT forest structure facilitated stable immobilization of substantial quantities of GOx on the rough electrode surface. The FTO-CNT/PEI/GOx electrode exhibited a sensitivity of 63.38 μA mM^−1^ cm^−2^ with a wide linear range of 70–700 µM glucose. Moreover, a synergetic effect generated by SWCNTs, GO (rGO), and cobalt phthalocyanine (CoPc) facilitated the creation of a SWCNT/rGO/CoPc GCE for the nonenzymatic detection of glucose in saliva, presenting a sensitivity of 992.4 μA mM^−1^ cm^−2^ and specificity for glucose in a complex interference environment [179] (Fig. 6F).

In the arena of diaper-based sensors, which have attracted considerable attention, there is still scant exploration of biomolecular strategies for in situ urine sensing, a deficiency that hampers the collection of health-evaluation information from the user’s urine. Li et al. [180] introduced a smart diaper that employs integrated multiplex electrochemical sensors (MECSs) for in situ urine analysis, selectively monitoring glucose levels. The electrode arrays embedded in the mechanically flexible diaper were tailored with CNT coatings and other chemicals, such as ion-selective membranes, enzymes, and Pt NPs, to scrutinize the target biomarkers correlated with urine (Fig. 6G). Furthermore, MECSs may be fashioned into prototypes, comprising a flexible circuit board and a Bluetooth signal transmitter, serving as an alternative means of bedside monitoring for patients, infants, and elderly individuals. Wang et al. [181] developed an innovative multi-calibrated glucose potentiometric (MCGP) sensing array, fusing a glucose electrode group, a pH electrode group, and a reference electrode channel. The array, containing a PtAu/CNT nanozyme modified with diboronic acid molecules, represents a pretreatment-free approach to evaluate glucose levels in human urine samples, exhibiting significantly enhanced selectivity for glucose. This study describes a novel technique for analyzing intricate samples and promoting home health monitoring. A comprehensive overview of certain other such sensors is depicted in Table 4.

## Conclusion: summary, prospects, and challenges

In recent years, CNTs have been widely used for glucose detection because of their huge specific surface area, strong absorption capacity and superior electron transport capability. Here, we review the recent advances in CNT-based electrochemical sensors. Notably, this review highlights the current progress, challenges and future directions of electrochemical glucose sensors and briefly forecasts the future direct employment in body fluids.

### Summary

In conclusion, this review has provided a comprehensive exploration of the design, manufacturing advancements, material synergies, and challenges associated with CNT-based electrochemical glucose sensors. Rapid progress in nanotechnology, microfluidics, miniaturization, and point-of-care sensing technology has spurred the development of sensitive, cost-effective, and user-friendly glucose monitoring tools. Researchers are actively integrating material design innovations, such as implantable and wearable microelectrodes and interstitial microneedles, to create a diverse array of adaptable CNT-based devices for biomarker detection in human biofluids. This review highlights the role of CNTs in high-performance wearable biosensors due to their flexibility and sub-nanometer thickness (equivalent to skin curvature) and predicts future advances in the detection of low glucose concentrations in a variety of body fluids and the integration of sensors into portable and implantable devices.

### Challenges and improvements in CNT-based glucose sensors

The development and utilization of CNT-based glucose sensors come with several challenges and opportunities for improvement in various aspects of design, performance, and safety.

#### Heterogeneity of SWCNTs

One of the primary challenges is the heterogeneity of SWCNTs. The availability of pure, semiconducting SWCNTs is essential for electronic device applications. Techniques such as density gradient ultracentrifugation, dielectric electrophoresis, and surfactant dispersion are used for SWCNT dispersion. However, these methods often involve complex and tedious separation processes, impeding their widespread use [9].

#### Catalytic synergy

CNTs cannot directly catalyze glucose reactions. Hence, they require combination with other catalytic materials to enhance performance. Initially, enzymatic CNT-based sensors faced limitations due to sensitivity to ambient temperature and pH, restricting their broad applications. Researchers have substituted enzymes with metals, metal oxide compounds, alloys, MOFs, and conducting polymers (CPs) to enhance catalytic efficiency and practicality under various conditions. Effective synergy between CNTs and metals is crucial for optimal sensor performance.

#### Selectivity and stability of nonenzymatic sensors

While metal-based nonenzymatic sensors offer better stability and broader environmental suitability, they are susceptible to interference from oxidation intermediates and high chloride ion concentrations. Transition metal-based sensors are typically designed for alkaline environments, which contrast with the neutral environment of blood. Noble metals such as gold can function in diverse pH conditions but are expensive. Enhanced selectivity of nonenzymatic sensors integrated with metals and CNTs is essential, and molecular- or atomic-scale interactions should be explored for improved glucose monitoring properties.

#### Performance of CP nanomaterials

CP nanomaterials are commonly used in wearable glucose sensors due to their flexibility. However, issues such as poor selectivity, adsorption of intermediates, surface poisoning, and low sensitivity limit their application. The incorporation of CNTs with other nanomaterials can synergistically enhance the overall performance of CP-based sensors.

#### Improvement of flexibility and biocompatibility

The future direction of implantable biosensors is toward flexible and microfiber structures. CNT fibers are suitable for implantation due to their stable binding and compatibility with tissue [183, 184]. However, the inherent softness of CNT fibers can limit direct implantation [185]. Modification of fiber probes with polymer hydrogels with variable elastic modulus can enhance biocompatibility and minimize tissue damage during implantation.

#### Wearable sensor applications

In wearable sensor applications, CNTs are preferred for their excellent conductivity and flexibility. Fabric sensors offer advantages in terms of comfort, breathability, and durability compared to thin-film sensors. The development of multiplexed assays for diabetes-related markers using CNTs as platforms shows promise for comprehensive biosensing applications [186]. Flexible CNT fiber-based platforms can be integrated into textiles for real-time health monitoring, providing consistent and robust sensing capabilities [187].

#### The toxicity of CNTs

Future research should focus not only on the detection range but also on biocompatibility, stability, durability, and real-sample analysis to ensure practical usability. The controversy over the toxicity of CNTs requires careful consideration, especially in direct contact scenarios such as skin and lung exposure [188, 189].

### Applications of CNT-based electrochemical glucose sensors for potential biological fluids

Owing to the demand for more convenient, diverse, and regionalized methods of detecting blood glucose, along with a need for rapid glucose response detection, the development and discussion of CNT-based electrochemical glucose sensors for multiple applications in various body fluids—including tears, exhaled breath condensate (EBC), nasal fluids, cerebrospinal fluid (CSF), and peritoneal fluid—are both necessary and meritorious.

While studies have brought to fruition CNT-based electrochemical glucose sensors for the detection of tear samples, the in-situ detection of tear glucose leveraging CNTs remains an unexplored territory. Properly prepared CNT films can attain a fusion of flexibility, light transmission, and electrical conductivity, aligning with the developmental trajectory of tear sensors [190]. Nonetheless, to date, tear sensors employing CNT films as substrates have not been found, a gap we perceive as a promising area of application.

EBC serves as a safe, noninvasive means for sampling fluids from the lower respiratory tract [191]. Since a primary challenge for EBC glucose sensors lies in the submicromolar sensitivity required [192, 193], sensors that integrate highly conductive CNTs into the EBC glucose sensing apparatus are ideally positioned to rectify such problems. Additionally, enzymatic sensors relying on H_2_O_2_ for indirect glucose content detection exhibit instability in EBC detection. If CNTs are employed to augment the performance of enzymatic glucose sensors, emphasis must be placed on enhancing enzyme stability. Similarly, respiratory fluids such as nasal fluid—which has not yet been definitively linked with blood glucose levels—are a novel and intriguing prospect in noninvasive glucose detection, even though electrochemical detection in nasal fluid remains undeveloped.

Furthermore, minimally invasive CNT-based glucose sensors for CSF and peritoneal fluids currently represent an uncharted area, and yet, they hold potential for future applications. Although detection in CSF is feasible under traumatic conditions, the pursuit of minimally invasive methods is still a promising avenue. To this point, implantable fiber biosensors based on CNTs have been designed to detect dopamine by probing deep brain tissue, but attempts to utilize electrochemical glucose sensors for CSF detection have yet to be made. Nonetheless, optimism prevails in the glucose detection domain. Importantly, no definitive evidence has been discovered regarding the toxicity of neural-related electrodes and biosensors founded on CNTs, and some reports even suggest that certain CNTs may promote cell growth [194, 195]. These findings may signify that CNTs are exceptionally promising materials for neural electrodes. Additionally, given the latency issues in subcutaneous continuous glucose monitoring (CGM), there have been proposals to utilize the intraperitoneal (IP) space for CGM [196]. Although the application of electrochemical glucose detection for IP is still in its infancy, the future likely holds promise for CNT-based electrochemical glucose sensors in IP applications, especially since CNTs can serve as flexible materials apt for long-term in vivo implantable detection.

It is vital to underscore that whether CNTs are employed as a fiber implantation material or as a dopant, careful consideration must be given to the in vivo safety of CNTs. This aspect should not be overlooked, even as strides are made to enhance sensor performance.

### The promising applications of CNT-based glucose sensors in the field of biomedical sensing

Nanotechnology has emerged as a focal point in diverse biomedical applications, including cancer therapy, owing to its capacity to manipulate materials within the size range of 1–1000 nm [197, 198]. CNTs are perceived as suitable candidates for cancer therapy due to their unique structural, mechanical, electrical, and thermal properties (often referred to as PTT) [199]. The extensive surface area of CNTs facilitates the loading of high concentrations of anticancer therapeutics, either through the utilization of disulfides as linkers or via adsorption. Furthermore, controlled drug delivery can be orchestrated by modifying CNTs with stimuli-responsive materials [200].

Subsequently, a design paradigm has been postulated for a nanorobot capable of navigation, cancer cell detection in the bloodstream, and precise drug delivery [201]. By exploiting glucose hunger-based cancer detectors immobilized on CNT sensors, a decrease in electrical resistance occurs upon binding to cancer cells. This phenomenon triggers an electric current that activates a nanoelectromechanical relay, or a mechanical transistor, breaching the containment chamber and thereby exposing an immune system-recognized drug to obliterate the cell. This concept heralds a transformative approach for CNT-based glucose sensors, extending beyond macroscopic glucose monitoring in humans to include the assessment of glucose levels in the microscopic environments of cancer cells. The integration of bionanosensing with sophisticated nanotransistor technology marks a promising frontier for in vivo medical diagnostics and therapy.

Furthermore, investigations have been conducted into the skin permeability of CNTs for the transdermal administration of therapeutic agents. However, findings reveal that CNTs alone are not permeable through the skin, giving rise to a dilemma: noninvasive wearable CNT-based electrochemical glucose sensors are unable to concurrently function as drug application surfaces. Nevertheless, limited studies have demonstrated that lipid/polymer functionalization and ionic introduction can enhance the skin permeability of CNTs [202].

The collective evidence elucidated above galvanizes biomedical researchers to probe the capabilities of CNT-based glucose sensors in therapeutic contexts, a pursuit imbued with tremendous potential for unlocking novel milestones in biomedical sensing. In the foreseeable future, the reach of CNT-based electrochemical glucose sensors will likely transcend mere diagnostic sensing, with the incorporation of feedback mechanisms heralding a new era for therapeutic applications within diagnostic-therapeutic integrated devices.

## Data Availability

Not applicable.
